# Quantum Interference between Fundamentally Different Processes Is Enabled by Shaped Input Wavefunctions

**DOI:** 10.1002/advs.202205750

**Published:** 2023-02-03

**Authors:** Jeremy Lim, Suraj Kumar, Yee Sin Ang, Lay Kee Ang, Liang Jie Wong

**Affiliations:** ^1^ School of Electrical and Electronic Engineering Nanyang Technological University 50 Nanyang Avenue Singapore 639798 Singapore; ^2^ Science, Mathematics and Technology Singapore University of Technology and Design 8 Somapah Road Singapore 487372 Singapore

**Keywords:** light–matter interactions, nanophotonics, quantum interference, ultrafast optics, waveshaping

## Abstract

This work presents a general framework for quantum interference between processes that can involve different fundamental particles or quasi‐particles. This framework shows that shaping input wavefunctions is a versatile and powerful tool for producing and controlling quantum interference between distinguishable pathways, beyond previously explored quantum interference between indistinguishable pathways. Two examples of quantum interference enabled by shaping in interactions between free electrons, bound electrons, and photons are presented: i) the vanishing of the zero‐loss peak by destructive quantum interference when a shaped electron wavepacket couples to light, under conditions where the electron's zero‐loss peak otherwise dominates; ii) quantum interference between free electron and atomic (bound electron) spontaneous emission processes, which can be significant even when the free electron and atom are far apart, breaking the common notion that a free electron and an atom must be close by to significantly affect each other's processes. Conclusions show that emerging quantum wave‐shaping techniques unlock the door to greater versatility in light‐matter interactions and other quantum processes in general.

## Graphical abstract

In this work, a general framework is presented for quantum interference between processes that can involve different fundamental particles or quasi‐particles. Shaping input wavefunctions is revealed to be a versatile and powerful tool for producing and controlling quantum interference between distinguishable pathways, beyond previously explored quantum interference between indistinguishable pathways.

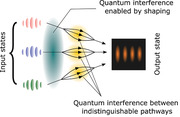

## 1 Introduction

Interest in controlling quantum processes has led people to seek increasingly precise ways of manipulating the wavefunctions involved—a process known as wave‐shaping. Through wave‐shaping, many unique wave patterns for particles including photons and electrons have been realized by introducing a well defined phase relation between different eigenstates. Shaped free electron wavepackets,^[^ ^1^, ^2^, ^3^, ^4^, ^5^, ^6^, ^7^, ^8^, ^9^, ^10^, ^11^, ^12^, ^13^, ^14^, ^15^, ^16^, ^17^, ^18^, ^19^, ^20^, ^21^, ^22^, ^23^, ^24^, ^25^, ^26^, ^27^ ^]^ for instance, are useful as probes of light–matter excitations for example, electron energy‐loss spectroscopy and its variants,^[^ ^8^, ^12^, ^13^, ^28^, ^29^, ^30^, ^31^, ^32^, ^33^, ^34^, ^35^, ^36^, ^37^ ^]^ cathodoluminescence microscopy,^[^ ^12^, ^13^, ^29^, ^38^ ^]^ and photon‐induced near‐field electron microscopy (PINEM),^[^ ^1^, ^2^, ^4^, ^5^, ^6^, ^7^, ^8^, ^9^, ^10^, ^11^, ^12^, ^13^ ^]^ etc.; and also as a means of tailoring light emission from free electrons.^[^ ^38^, ^39^, ^40^, ^41^, ^42^, ^43^, ^44^, ^45^, ^46^, ^47^, ^48^, ^49^, ^50^ ^]^ Likewise, shaped bound electron (e.g., atomic states) form the basis of many fields including quantum metrology,^[^ ^51^, ^52^, ^53^, ^54^, ^55^ ^]^ quantum information technologies,^[^ ^56^, ^57^, ^58^, ^59^, ^60^, ^61^, ^62^, ^63^, ^64^ ^]^ quantum integrated circuits,^[^ ^65^, ^66^, ^67^, ^68^, ^69^ ^]^ and photon generation and manipulation.^[^ ^66^, ^70^, ^71^, ^72^, ^73^, ^74^, ^75^, ^76^ ^]^ Many techniques exist to shape photons and quasi‐particles like polaritons.^[^ ^77^, ^78^, ^79^, ^80^, ^81^, ^82^, ^83^, ^84^, ^85^, ^86^, ^87^ ^]^ Recently, shaped neutron wavefunctions—especially twisted states—have garnered interest as possible probes for nuclear structure and interactions,^[^ ^88^, ^89^, ^90^ ^]^ and for neutron interferometry and optics.^[^ ^91^, ^92^, ^93^, ^94^, ^95^ ^]^

The study of interference effects in quantum systems dates back to as far as the Davisson–Germer experiment where it was used to prove the wave nature of free electrons.^[^ ^96^ ^]^ The discovery of the wave nature of the electron proved the wave‐particle duality hypothesis advanced by de Broglie, which can be explained through the framework of quantum mechanics. Given multiple transition pathways between an initial quantum state and a final quantum state of a system, there will in general be interference between the pathways—these pathways are different yet indistinguishable. For example, recent research into quantum interference between indistinguishable pathways in electron–light interactions has proven useful in realizing and manipulating ultrafast electron wavepackets.^[^ ^4^, ^97^ ^]^ This raises the fundamental question of whether quantum interference between distinguishable pathways is also possible.

Here, we show that quantum interference between different and distinguishable pathways is indeed possible with the use of shaped input wavefunctions. Specifically, we present a general framework for quantum interference between arbitrary types and numbers of quantum systems, enabled by shaped input wavefunctions. We see that wave‐shaping results in quantum interference beyond just two processes, leading to a dominance of quantum interference effects as the number of systems with shaped input wavefunctions increases. We show that quantum interference between distinguishable pathways can be controlled by tailoring the input wavefunctions of the particles or quasi‐particles involved.

We present two examples of quantum interference between distinguishable pathways, enabled by electron wave‐shaping. These examples involve interactions between free electrons, bound electrons, and light. In the first example, we show that quantum interference can eliminate the zero‐loss peak of the output electron spectrum in free‐electron–light interactions at moderate coupling strengths. We also show that quantum interference can dramatically enhance or suppress the other peaks in the free electron gain/loss spectrum. Our results are achievable with parameters well within the capabilities of current PINEM setups. In the second example, we show that quantum interference via wave‐shaping can occur between free electron and atomic (also known as bound electron) spontaneous emission processes. We choose the second example for the following reasons: i) There has been much excitement and progress in shaping electron wavepackets both spatially and temporally;^[^ ^1^, ^2^, ^3^, ^4^, ^5^, ^6^, ^7^, ^8^, ^9^, ^10^, ^11^, ^12^, ^13^, ^14^, ^15^, ^16^, ^17^, ^18^, ^19^, ^20^, ^21^, ^22^, ^23^, ^24^, ^25^, ^26^, ^27^, ^98^ ^]^ ii) the quantum interference enabled between bound electron processes and free electron processes, such as atomic and free electron spontaneous emission, has never been explored; iii) free electron and bound electron spontaneous emission processes are not expected to significantly influence each other unless the free electron and the bound electron are very close by. The requirement for the free electron and the bound electron to be close to directly interact with each other is due to the near‐field nature of Coulomb interactions.^[^ ^99^, ^100^, ^101^, ^102^, ^103^, ^104^ ^]^ On the contrary, we find that owing to quantum interference, shaped free electrons and shaped bound electrons can affect each other even when both systems are physically far apart. Our results show that maximum enhancement or suppression of spontaneous emission can be achieved over a wide range of free electron kinetic energies (e.g., 100 eV to 1 MeV) and emission frequencies (e.g., optical to terahertz). Our findings fill an important gap in the understanding of quantum interference. Our work also motivates the development of shaping techniques for a wider variety of quantum systems. Quantum interference between distinguishable pathways can be used to leverage the full potential of quantum interference for on‐demand tailoring of quantum processes in light–matter interactions and beyond.

## 2 Results

### 2.1 General Framework for Quantum Interference between Distinguishable Pathways

Consider a collection of *N* distinct systems, for example, free electrons, bound electrons, photons, atomic nuclei, neutrons, and any other fundamental particles or quasi‐particles. We denote the eigenstates of the *j*th system as $|\alpha_{j}\rangle$ with corresponding eigenvalues $\alpha_{j}$. We consider an initial state of the form $\left|\text{initial}\right\rangle ={⨂}_{j=1}^{N}\left(\sum_{\alpha_{j}}C_{\alpha_{j}}|\alpha_{j}\rangle \right)$, where $|C_{\alpha_{j}}{|}^{2}$ is the probability of finding the *j*th system in $|\alpha_{j}\rangle$. The probability that |initial⟩ scatters into a final state $\left|\text{final}\right\rangle =|\beta_{1},\text{…},\beta_{N}\rangle$ after an arbitrary interaction is described by the scattering operator $\hat{S}$ which is given by $P_{\text{final}}=\left|\langle \text{final}\right|\hat{S}\left|\text{initial}\right\rangle {|}^{2}$. Expanding |initial⟩ in full and defining the coherence and population of the *j*th system as $\rho_{\alpha_{j}\alpha_{j}^{\prime }}\equiv C_{\alpha_{j}}{\bar{C}}_{\alpha_{j}^{\prime }}$ (where $\alpha_{j}\neq \alpha_{j}^{\prime }$) and $p_{\alpha_{j}}\equiv \rho_{\alpha_{j}\alpha_{j}}$ (overbars denote complex conjugates) respectively, we express $P_{\text{final}}$ as

(1) Pfinal=∑α1,…,αNpα1…pαNSα1,…,αNfinal2︷without quantum interference+[N1terms of∑all exceptαj∑αj≠αj′ραjαj′pα1…pαNpαjS…,αj,…finalS¯…,αj′,…final]︷1-process quantum interference+[N2terms of∑all exceptαj,αk∑αj≠αj′αk≠αk′ραjαj′ραkαk′pα1…pαNpαjpαkS…,αj,αk,…finalS¯…,αj′,αk′,…final]︸2-process quantum interference+[N3terms of∑all exceptαj,αk,αl∑αj≠αj′αk≠αk′αl≠αl′ραjαj′ραkαk′ραlαl′pα1…pαNpαjpαkpαlS…,αj,αk,αl,…finalS¯…,αj′,αk′,αl′,…final]︸3-process quantum interference+⋯+[∑α1≠α1′,…,αN≠αN′ρα1α1′⋯ραNαN′Sα1,…,αNfinalS¯α1′,…,αN′final]︸N-process quantum interference,

where $S_{\alpha_{1},\text{…},\alpha_{N}}^{\text{final}}\equiv \left\langle \text{final}\right|\hat{S}\left|\alpha_{1},..,\alpha_{N}\right\rangle$ is the scattering matrix element. The total scattering probability into states that share the same final values of quantum numbers $\beta_{m},\text{…},\beta_{n}$ is $P_{\beta_{m},\text{…},\beta_{n}}=\sum_{\text{all except}\beta_{m},\text{…},\beta_{n}}P_{\text{final}}$. The first term of Equation (1) is the total probability in the absence of quantum interference between distinguishable pathways due to wave‐shaping. Note that the absence of quantum interference due to wave‐shaping corresponds to the scenario where multiple eigenstates exist, but they are related by random phases, resulting in quantum interference disappearing upon statistical averaging. The terms in the *R*th square parentheses, where $R\in Z^{+}$, contain the NR possible quantum interference terms that can arise between *R* of the *N* systems. Crucially, our framework reveals the importance of shaped input wavefunctions as a means to tailor quantum interference between distinguishable pathways: the initial wavefunctions of the systems participating in quantum interference must be a superposition of eigenstates with well‐defined phase relations between them. The shaping of input states ensures that the coherences of the systems involved in the quantum interference—and hence the relevant quantum interference terms in Equation (1)—are non‐zero upon statistical averaging.

**Figure 1:**
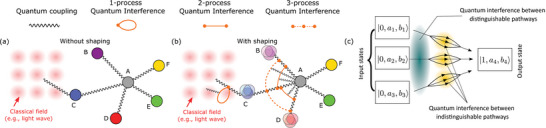
A general framework for quantum interference enabled by shaped input quantum wavefunctions, providing a means to tailor quantum processes via quantum wave‐shaping. To illustrate this, we consider the example of five systems (B–F) coupled to system A. a) When systems B to F have unshaped (i.e., either single‐input state or a superposition of eigenstates with random phase relations, represented by solid circles) input wavefunctions, quantum interference between distinguishable pathways is absent and the total contribution to any process between system A and the other systems is simply the sum of the individual contributions arising from the direct coupling between system A and the other systems considered in isolation. We note here that the quantum coupling indicated by the squiggly line includes quantum interference between indistinguishable yet different pathways for the two systems being coupled. b) When some of the systems (B–D here) have shaped input wavefunctions (i.e., superposition of eigenstates with fixed phase relations, denoted by overlapping translucent circles), quantum interference between distinguishable pathways occurs between the processes associated with the shaped wavefunctions, resulting in additional contributions. Notably, quantum interference can involve more than two processes in general, resulting in a dominance in the number of quantum interference contributions as the number of systems with shaped wavefunctions increases. Note that 1‐process quantum interference can occur for instance, when the scattering events involve classical fields (e.g., self‐loop in coupling between system C and classical field). c) We illustrate the difference between quantum interference between indistinguishable yet different pathways and the quantum interference due to wave‐shaping that we study here. Consider a scenario where we have two different particles/quasi‐particles A and B as two systems. Let them be coupled to some other particle/quasi‐particle C which has the possible quantum states |0〉 and |1〉. We can then see that for some input state of the system that can transition to a final state, there exist multiple transition pathways in general. Note that these paths are indistinguishable as one cannot tell which path the transition takes place through, yet they are different nonetheless and these paths can interfere. If we shape the initial states of particles A and B, we see that in general, multiple input eigenstates that make up the initial state of the combined system transition to the same output state. Note that these transition pathways are distinguishable (and different) since they originate from different initial states. These transitions between different input states to the same output states will interfere, causing quantum interference, which can be traced back to wave‐shaping since wave‐shaping implies the presence of multiple states. This type of quantum interference is absent in the unshaped case (due to having only one input state). Quantum interference between distinguishable pathways is complementary to quantum interference between indistinguishable pathways since both can occur at the same time as illustrated above.

**Figure** **1** illustrates a system A coupled in a pairwise manner to systems B to F (note in general that Equation (1) is not limited to pairwise coupling). We see that shaping the input wavefunctions of systems B to D results in 2‐process and 3‐process quantum interference between the processes associated with these shaped wavefunctions. This quantum interference provides additional contributions to the interactions of the other particles with A. 1‐process quantum interference can arise, for instance, when a system C with its input wavefunction shaped couples to classical fields (e.g., a light wave).

### 2.2 Elimination of Zero‐Loss Peak by Quantum Interference in Free‐Electron‐Light Interactions

**Figure 2:**
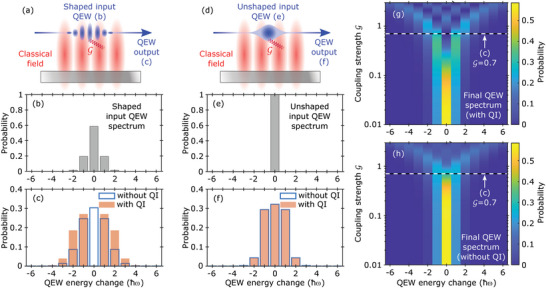
Quantum interference in free‐electron‐light interactions at moderate coupling strengths, resulting in elimination of the zero‐loss peak by destructive quantum interference, and enhancement of satellite peaks by constructive quantum interference. a) An incoming shaped free electron (modeled as a quantum electron wavepacket (QEW)), with input spectrum shown in (b), scatters off a classical light field with a dimensionless coupling strength G=0.7, resulting in an output electron spectrum where the zero‐loss peak completely vanishes. If quantum interference contributions are neglected (unfilled bars with blue outlines in (c)), which occurs, for instance, when there is no fixed phase relation between the input eigenstates, the zero‐loss peak dominates. d) In contrast, for an unshaped incoming QEW (input spectrum shown in (e)), the output QEW spectrum in the presence and absence of quantum interference coincide (as shown in (f)), which implies that quantum interference contributions vanish for unshaped input QEWs. (g,h) compare the output QEW spectrum in the presence and absence of quantum interference, respectively, as a function of G. The contribution of quantum interference is already substantial even for weaker interactions of about G≈0.1. As shown in (h), the complete suppression of gain/loss peaks away from the zero‐loss peak can be achieved for coupling strengths G≳1.

Consider a shaped incoming free electron, that is, a quantum electron wavepacket (QEW), being scattered by a classical light wave at moderate coupling strengths (**Figure** **2**a,b). The output energy gain/loss spectrum (orange bars in Figure 2c) shows a complete suppression of the zero‐loss peak—a direct consequence of quantum interference. If quantum interference is neglected the zero‐loss peak remains dominant in the output spectrum (blue‐outlined unfilled bars in Figure 2c). Note that neglecting quantum interference corresponds to the physical scenario where a random phase relation exists between the input electron eigenstates, which results in the disappearance of quantum interference effects upon statistical averaging. In addition to the complete suppression of the zero‐loss peak, quantum interference also enhances the gain/loss peaks away from the zero‐loss peak. In contrast, for an unshaped incoming QEW (Figure 2d,e), there are no quantum interference contributions and the zero‐loss peak dominates in the output spectrum (Figure 2f). We model the wavefunction of the incoming QEW with the general form $\left|\mathrm{initial}\right\rangle =\sum_{n}C_{n}\left|n\right\rangle$, where $C_{n}=e^{i\phi_{\mathrm{mod}}}J_{n}\left(2\right|G_{\mathrm{mod}}\left|\right)$ is the initial complex amplitude of the *n*th energy gain/loss peak of the incoming QEW,^[^ ^4^ ^]^ $\phi_{\mathrm{mod}}$ is the modulation phase ($\phi_{\mathrm{mod}}=0$), $J_{n}$ is the Bessel function of the first kind, and $G_{\mathrm{mod}}$ is the dimensionless coupling strength of the shaping stage ($G_{\mathrm{mod}}=0.5$ in Figure 2a–c,g,h and $G_{\mathrm{mod}}=0$ in Figure 2d–f). The exact QEW‐light interaction is described by the scattering operator $\hat{S}\equiv \exp\left(G^{*}\hat{b}-G{\hat{b}}^{†}\right)$,^[^ ^4^ ^]^ where G is the dimensionless coupling strength between the incoming QEW and the classical light field. The coupling strength G is dependent on the velocity of the free electron and also the spatio‐temporal structure of the light field. It follows that different velocities of the free electron could correspond to the same coupling strength provided the light field is structured differently. Here, $\hat{b}$ (${\hat{b}}^{†}$) decrements (increments) each QEW eigenstate by a unit photon energy. We obtain the *N*th energy gain/loss peak of the final output QEW probability spectrum (i.e., the probability that |initial⟩ scatters into a final state |N⟩) as

(2) $$\begin{array}{c} \begin{array}{c} P_{N}=\underset{\text{without quantum interference}}{\underset{︸}{\sum_{n}|C_{n}{|}^{2}{\left|\left\langle N|\hat{S}|n\right\rangle \right|}^{2}}}+\underset{\text{1-process quantum interference}}{\underset{︸}{\sum_{m\neq n}C_{m}C_{n}^{*}\left\langle N|\hat{S}|m\right\rangle {\left\langle N|\hat{S}|n\right\rangle }^{*}}},\;m,n,N\in Z \end{array} \end{array}$$

which is the sum of the first and second terms in Equation (1) with only a single system (the QEW) as input. In Figure 2a–f, we consider a coupling strength of G=0.7, which is well within the reach of existing PINEM setups, where coupling strengths on the order of G≈100 has been demonstrated.^[^ ^11^ ^]^ For smaller coupling strengths of G≳0.1, the zero‐loss peak appears but remains suppressed by destructive quantum interference, as we show in Figure 2g,h. Additionally, for G≳1, complete suppression of the gain/loss peaks away from the zero‐loss peak can also occur due to quantum interference. We find that our results still hold for incoming QEWs of different shapes (Section SI, Supporting Information).

### 2.3 Quantum Interference between Free Electron and Bound Electron Spontaneous Emission Processes

**Figure 3:**
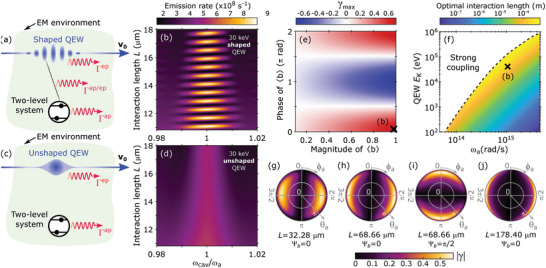
Quantum interference between distinguishable pathways provides a means to tailor spontaneous emission from free electrons and bound electrons via quantum waveshaping. a) Shaped quantum electron wavepackets (QEWs) and bound electrons (atomic two‐level system) within an electromagnetic (EM) environment separately emit photons at rates of Γ^ep^ and Γ^ap^, respectively. Quantum interference between these spontaneous emission processes (dotted line) results in a third emission process at rate $\Gamma^{\mathrm{ap}/\mathrm{ep}}$. This third emission process (the quantum interference term) can enhance or suppress the total spontaneous emission rate as a function of interaction length *L* (as shown in (b)) by more than 70%. c) In contrast, for unshaped incoming QEWs, quantum interference is absent (as shown in (d)) and the total spontaneous emission rate is $\Gamma^{\mathrm{ap}}+\Gamma^{\mathrm{ep}}$. Quantum interference can be tailored using the QEW and bound electron shapes, which are determined by bunching factor $\left\langle b\right\rangle =\left|\left\langle b\right\rangle \right|e^{i\Psi_{b}}$ and coherence $\rho_{\text{eg}}^{a}=\left|\rho_{\text{eg}}^{a}\right|e^{i\phi_{a}}$, respectively. Defining the figure of merit $\gamma =\Gamma^{\mathrm{ap}/\mathrm{ep}}$/($\Gamma^{\mathrm{ap}}+\Gamma^{\mathrm{ep}}$), which is a measure of the quantum interference contribution, we see from (e) that larger |$\gamma_{\max}$| = max(|γ|) is achieved for larger |⟨b⟩|, and that quantum interference can be tuned to enhance or suppress the spontaneous emission rate by controlling the phase of the bunching factor $\Psi_{b}$. (f) shows the dependence of the optimal length *L*_opt_—at which $\gamma_{\max}$ is achieved—as a function of QEW kinetic energy $E_{K}$ and bound electron emission frequency $\omega_{a}$. The polar plots in (g–j) show the value of |γ| on the Bloch sphere representing the initial shape of the bound electron system at various values of *L*. (h,i) show that the profile of |γ| on the initial Bloch sphere can be manipulated by varying $\Psi_{b}$. Unless otherwise stated, we consider a 30 keV shaped QEW with ⟨b⟩=0.99. Our two‐level, bound electron system is a Sn‐N vacancy with $\omega_{a}$ ≈ 3 × 10^15^ rad s^−1^ and transition dipole moment of $\left|d\right|=4.33\times 10^{-29}$ Cm aligned parallel to the field. For (g–j), the azimuthal angle $\phi_{a}$ is the phase of the bound electron coherence and θ_a_ is related to the excited state population through $\cos^{2}\left(\theta_{a}/2\right)$, and we use a 30 keV shaped QEW with ⟨b⟩=0.58.

We now apply our framework to study quantum interference between free electron and bound electron spontaneous emission processes in a cavity (or any electromagnetic environment in general). A shaped incoming QEW (**Figure** **3**a) can induce a quantum interference contribution to the spontaneous emission $\Gamma^{\mathrm{ap}/\mathrm{ep}}$ which enhances or suppresses the sum of the individual spontaneous emission processes $\Gamma^{\text{ap}}+\Gamma^{\text{ep}}$ by more than 70%, depending on interaction length *L* and cavity angular frequency $\omega_{\text{cav}}$ (Figure 3b). Here $\Gamma^{\text{ap}}$ and $\Gamma^{\text{ep}}$ denote the bound electron and free electron spontaneous emission rates, respectively. In contrast, there is no quantum interference for an unshaped (Gaussian) incoming QEW (Figure 3c,d). As expected, we find that the spontaneous emission rates peak sharply at resonance ($\omega_{\text{cav}}=\omega_{a}$). We consider the resonant case for the rest of the example. The incoming QEW of velocity $v=v_{0}\hat{z}$ has a central kinetic energy of $E_{K}$ = 30 keV. We treat the bound electron system as a two‐level atomic system by considering a tin‐vacancy (SnV) center^[^ ^105^ ^]^ of emission frequency $\omega_{a}$ ≈ 3 × 10^15^ rad s^−1^ and dipole moment $d=\hat{z}4.33\times 10^{-29}$ Cm (aligned parallel to the field).

Importantly, the shapes of the QEW and bound electron can be used to tailor $\Gamma^{\mathrm{ap}/\mathrm{ep}}$. We define the figure of merit $\gamma =\Gamma^{\mathrm{ap}/\mathrm{ep}}$/($\Gamma^{\mathrm{ap}}+\Gamma^{\mathrm{ep}}$) as a measure of quantum interference's relative contribution to the spontaneous emission rate. Figure 3e shows how the incoming QEW shape—determined by the bunching factor $\left\langle b\right\rangle =\left|\left\langle b\right\rangle \right|e^{i\Psi_{b}}$ – affects $\gamma_{\max}$, which is the maximum possible γ across all *L*. For the case shown in Figure 3d (black cross in Figure 3e), we considered ⟨b⟩=0.99, which has recently been shown to be feasible.^[^ ^15^ ^]^ Even for a more modest bunching factor of ⟨b⟩≈0.58, which is attainable using PINEM (see Section SII, Supporting Information and ref. [15]), for instance, |$\gamma_{\max}$| ≳ 0.4 can still be achieved. Varying the phase $\Psi_{b}$ (e.g., using phase of the modulating field) also allows us to control the amount by which the overall spontaneous emission rate is enhanced or suppressed. Figure 3f shows that the optimal interaction length *L*_opt_ needed to achieve |$\gamma_{\max}$| ≈ 0.707, which is on the order of ≈10 nm to ≈100 µm, falls in the range of experimentally realizable optical and terahertz cavity dimensions.^[^ ^106^, ^107^, ^108^, ^109^, ^110^, ^111^, ^112^, ^113^, ^114^ ^]^ Furthermore, the required QEW energies, which range from ≈100 eV to ≈1 MeV, are achievable using lab‐scale electron sources. Thus, it should be already feasible to perform experiments to observe quantum interference in spontaneous emission from superconducting qubits and quantum dots,^[^ ^115^, ^116^, ^117^, ^118^, ^119^, ^120^, ^121^, ^122^, ^123^, ^124^, ^125^ ^]^ which radiate in the terahertz and optical regimes (Section SIII, Supporting Information). An approximate analytical expression for *L*_opt_ is presented in Section SIV, Supporting Information.

The quantum interference contribution can also be controlled via the bound electron shape, which is determined by the coherence $\rho_{\text{eg}}^{a}=\left|\rho_{\text{eg}}^{a}\right|e^{i\phi_{a}}$ between its excited and ground states. We show the dependence of the quantum interference contribution on the atomic coherence in Figure 3g–j, which depicts |γ| as a function of the initial bound electron Bloch sphere at various values of *L*. Here, θ_a_ (radial coordinate) and $\phi_{a}$ (angular coordinate) are related to the excited state population $\rho_{\text{ee}}^{a}$ and coherence $\rho_{\text{eg}}^{a}$ through $\rho_{\text{ee}}^{a}=\cos^{2}\left(\theta_{a}/2\right)$ and $\rho_{\text{eg}}^{a}=\left(e^{i\phi_{a}}/2\right)\sin\theta_{a}$, respectively. The bunching factor phase $\Psi_{b}$ can be used to azimuthally rotate the profile of γ on the bound electron Bloch sphere, as seen in Figure 3h,i.

For Figure 3, we model the quantum interference contribution $\Gamma^{\mathrm{ap}/\mathrm{ep}}$ by considering a QEW of velocity $v=v_{0}\hat{z}$, modulated at frequency $\omega_{\mathrm{mod}}$ and bunching factor $\left\langle b\right\rangle =\left|\left\langle b\right\rangle \right|e^{i\Psi_{b}}$ passing through an electromagnetic (EM) environment (e.g., cavity, waveguide) of length *L* (also the interaction length) containing a bound electron system of coherence $\rho_{\text{eg}}^{a}=\left|\rho_{\text{eg}}^{a}\right|e^{i\phi_{a}}$. To first order in perturbation theory (i.e., weak coupling regime), we find that the single‐photon spontaneous emission rate arising from the quantum interference between the free electron and bound electron spontaneous emission processes is (Section SV, Supporting Information)

(3) $$\begin{array}{c} \Gamma^{\mathrm{ap}/\mathrm{ep}}=\frac{\tau }{\hbar }\frac{ev_{0}\omega_{a}\left|d\right|}{\varepsilon_{0}V\omega_{\mathrm{cav}}}|\rho_{\text{eg}}^{a}||\left\langle b\right\rangle |\cos\left(\xi \right)\mathrm{sinc}\left[\frac{(\omega_{\mathrm{cav}}-\omega_{a})\tau }{2}\right]\mathrm{sinc}\left[\frac{(\beta_{0}\omega_{\mathrm{cav}}-\omega_{\mathrm{mod}})\tau }{2}\right]\mathrm{sinc}\left[\frac{(\omega_{\mathrm{cav}}-\omega_{\mathrm{mod}})\tau }{2}\right] \end{array}$$

where the bound electron is located at $r=(0,0,z_{a})$. The EM environment supports a single dominant longitundinal field mode of angular frequency $\omega_{\text{cav}}$ and wavevector $q=(0,0,\omega_{\mathrm{cav}}/c)$, where *c* is the free‐space speed of light. Such a mode is realizable, for instance, using a racetrack waveguide.^[^ ^26^ ^]^ In Equation (3), ℏ is the reduced Planck constant, ϵ_0_ is the free‐space permittivity, *e* > 0 is the elementary charge, τ = *L*/*v*_0_ is the interaction duration, d is the bound electron transition dipole moment, *V* is the mode volume, *m*_e_ is the electron rest mass, β_0_ = *v*_0_/*c* is the normalized free electron velocity, ξ = $\phi_{a}$ − ($\omega_{\text{cav}}z_{a}$/*c*) − π/2 + $\Psi_{b}$, and $\phi_{a}$ is the bound electron coherence phase. The total spontaneous emission rate is simply Γ^ap^ + Γ^ep^ + $\Gamma^{\mathrm{ap}/\mathrm{ep}}$. Unless otherwise stated, the initial excited and ground state populations are equal, corresponding to coherence magnitude $|\rho_{\text{eg}}^{a}|=1/2$. We set $\phi_{a}$ − ($\omega_{\text{cav}}z_{a}$/*c*) = π/2, which maximizes the contribution of $\Gamma^{\mathrm{ap}/\mathrm{ep}}$. Note that Γ^ap^ +Γ^ep^ and $\Gamma^{\mathrm{ap}/\mathrm{ep}}$ are derived from the first and third terms of Equation (1) respectively when three systems (QEW, bound electron, and photon) are considered and the final state is summed over all possible output states containing 1 photon. The second term in Equation (1) (1‐process quantum interference) vanishes. Importantly, we see from Equation (3) that if either the free electron state or bound electron state (or both) is unshaped (corresponding to ⟨b⟩ = 0 and $\rho_{\text{eg}}^{a}=0$, respectively), $\Gamma^{\mathrm{ap}/\mathrm{ep}}$ = 0 and the quantum interference contribution vanishes, which we expect.

In our specific example of quantum interference between free electron and bound electron spontaneous emission processes, we find that quantum interference affects the total spontaneous emission rate substantially even when the free electron and bound electron systems are physically far apart. On the other hand, the Coulomb interaction between the atomic system and the QEW relies on the proximity between the atom and the QEW and has been leveraged in free‐electron‐bound‐electron‐resonant interaction to encode information on bound electron coherence and dephasing in electron spectra.^[^ ^99^, ^100^, ^101^, ^102^, ^103^, ^104^ ^]^ Thus, our work provides a complementary route towards free‐electron quantum metrology without the requirement of the bound electron system and QEW being physically near each other.

## 3 Discussion

In essence, the far‐reaching implications of our general quantum interference framework are as follows: i) Fundamentally distinct quantum processes can be made to affect each other through quantum interference by shaping the input wavefunctions; ii) in the presence of multiple shaped wavefunctions, multiple types of quantum interference can arise, which can lead to dominance in the number of quantum interference terms in the overall output rate; iii) quantum interference can exist not only between quantum systems, but also between quantum systems and classical fields. These quantum interference‐driven effects are enabled by shaped wavefunctions—a fundamental tool revealed by our general framework. Our framework provides the connection between shaped input wavefunctions and quantum interference between distinguishable pathways, showing that the former is a prerequisite for the latter. The framework also motivates the development of innovative shaping techniques for fundamental particles and other quantum systems. Neutrons, for instance, with their ability to couple to all four fundamental forces, hypothetical particles (e.g., dark matter, axions), and interactions (e.g., modified gravity),^[^ ^126^ ^]^ are promising testbeds for the foundations of cosmology and quantum mechanics. Ongoing efforts to shape the neutron imply that it may soon be possible to observe quantum interference between neutron‐driven processes and other types of processes.^[^ ^91^, ^94^ ^]^ Thus, quantum interference between distinguishable pathways provides additional degrees of freedom through which exotic interactions and particles can be probed.

While we have only considered unentangled, that is, pure input quantum states, our framework can also accommodate entangled, that is, mixed input quantum states by using a more general expression for the input state instead of that used in Equation (1). The new expression for the input state is given by $\left|\text{initial}\right\rangle =\sum_{\alpha_{1},\text{…},\alpha_{N}}C_{\alpha_{1},\text{…},\alpha_{N}}|\alpha_{1}\rangle ⊗\text{…}⊗|\alpha_{N}\rangle$, where $C_{\alpha_{1},\text{…},\alpha_{N}}\neq C_{\alpha_{1}}\text{…}C_{\alpha_{N}}$ in general. Thus, our framework can be used to study the effects of entangled input states, opening up a rich field of exploration. Our findings also suggest exciting prospects for applying our framework to processes that go beyond controlling photon emission. For instance, the interference between free‐electron‐photon and free‐electron‐bound‐electron interactions for manipulation of free electron wavepackets and photon statistics. Similarly the interference between free‐electron‐bound‐electron and bound‐electron‐photon interactions for manipulation of bound electron population and coherence.

The degree to which quantum interference contributes depends on how the electrons are shaped, which is reflected by the modulation coupling strength $G_{\mathrm{mod}}$ in the case of the free‐electron‐classical light example. $G_{\mathrm{mod}}$ depends on the velocity of the free electron and the intensity of the light field used to shape the electron. Figure S6, Supporting Information, shows the trend in quantum interference quantified by a figure of merit for increasing $G_{\mathrm{mod}}$. For the free‐electron‐bound‐electron‐light example, the relevant shaping parameters include the bunching factor of the free electron and the bound electron coherence $\rho_{\text{eg}}^{a}$. In particular, the bunching factor magnitude |⟨b⟩| should be as close to the theoretical maximum of 1 as possible. Moreover the phase of the bunching factor should allow for the cosine term in Equation (3) to be 1. These dependencies have been explored in Figure 3e. The interaction length can also determine the degree of quantum interference observed. We have also plotted the optimal interaction length required to maximize the quantum interference contribution as a function of both the electron kinetic energy and the angular frequency of the two‐level system band‐gap $\omega_{a}$ in Figure 3f and Figure S3, Supporting Information, respectively. We note that the required free electron velocity for zero‐loss peak suppression in our first example can take on multiple values as it is dependent on the modulation term $G_{\mathrm{mod}}$.

We mention briefly here the matter of momentum matching for both the examples considered. From Equation (S27), Supporting Information, describing the electron–photon coupling, we see that in the limit of long interaction time (corresponding to large *L*), the momentum matching condition should be $\Delta k=\omega_{\mathrm{cav}}/c$. However, for finite interaction times, the electron still couples to the cavity mode but the coupling strength is weaker and proportional to $\mathrm{sinc}(\frac{L}{2}\left(\Delta k-\omega_{\mathrm{cav}}/c\right))$. Systems like ours where free electrons interact with single cavity mode radiation have also been considered in previous works, where the momentum matching condition was also dealt with in the same way as above.^[^ ^26^ ^]^

The general framework for quantum interference we present also subsumes phenomena like electromagnetically induced transparency,^[^ ^127^, ^128^ ^]^ where destructive quantum interference between transition amplitudes in a three‐level system renders the system transparent in a spectral window, as well as weakly coupled free‐electron–photon interactions,^[^ ^45^, ^129^ ^]^ where quantum interference is analyzed as mixed‐order terms arising from the interference between orders of a perturbative series expansion. Our framework goes far beyond the prediction of these phenomena, as we show through two examples of quantum interference between distinguishable pathways, made possible by the wave‐shaping of particle wavefunctions: the vanishing of zero‐loss peak in electron–light interactions for moderate coupling strengths, and quantum interference between free electrons and bound electron spontaneous emission processes. Unlike any other existing framework, our framework shows that shaped wavefunctions are a powerful tool for enabling quantum interference between distinguishable pathways involving fundamentally different processes.

## 4 Conclusion

In summary, we present a general framework showing that shaped input wavefunctions enables quantum interference between distinguishable pathways, for processes that involve arbitrary types and numbers of particles/quasi‐particles enabled by shaped input wavefunctions. Intriguingly, we find that quantum interference between more than two processes is possible, leading to a dominance in the number of quantum interference terms as the number of shaped input wavefunctions increases. We present two examples of quantum interference between distinguishable pathways, enabled by electron wave‐shaping. In the first example, we show using experimentally realistic parameters that quantum interference can eliminate the zero‐loss peak of the output free electron spectrum in free‐electron‐light interactions at moderate coupling strengths. We also show that quantum interference can dramatically enhance or suppress the satellite peaks in the output free electron spectrum. Such spectral control potentially gives us extra versatility in free‐electron‐light interactions (e.g., PINEM), which have been widely studied for its applications in spatio‐temporal electron imaging of nanoscale particles and in wave‐shaping ultrafast electrons. In the second example, we show that quantum interference can occur between shaped free electron and bound electron spontaneous emission processes even when both systems are physically distant and not able to interact via the Coulomb force. We find that the total spontaneous emission rate can be enhanced or suppressed by up to 70% relative to the sum of isolated free electron and bound electron spontaneous emission rates as a direct consequence of quantum interference. Quantum interference between distinguishable pathways thus provides an additional means of controlling spontaneous emission, a process fundamental to a wide range of applications, from scintillation and single‐photon generation to the X‐ray emission in synchrotrons and free electron lasers. Our findings fill an important gap in the understanding of wave‐shaping as a versatile tool to create and control quantum interference between distinguishable pathways, and to introduce unexplored methods of tailoring and optimizing quantum interactions. Coupled with growing interest in shaping a wide range of quantum systems, including free electrons and neutrons, our work unlocks the possibility of quantum interference for on‐demand tailoring of light‐matter interactions and beyond.

## Conflict of Interest

The authors declare no conflict of interest.

## Supporting information

Supporting Information

## Data Availability

The data that support the findings of this study are available from the corresponding author upon reasonable request.
