# Supplementary material for: Quantum Interference between Fundamentally Different Processes Is Enabled by Shaped Input Wavefunctions
Source: Adv Sci (Weinh). 2023 Feb 3;10(10):2205750. doi: 10.1002/advs.202205750 (PMC10074114; doi:10.1002/advs.202205750)
Supplement: Supplementary file 1 — Supporting Information [file ADVS-10-2205750-s001.pdf]

ADVANCED SCIENCE
Open Access

Supporting Information

for *Adv. Sci.*, DOI 10.1002/advs.202205750

Quantum Interference between Fundamentally Different Processes Is Enabled by Shaped Input Wavefunctions

*Jeremy Lim, Suraj Kumar, Yee Sin Ang, Lay Kee Ang and Liang Jie Wong**

Supplemental Material: Quantum interference between fundamentally different processes is enabled by shaped input wavefunctions

Dr. Jeremy Lim,[1] Mr. Suraj Kumar,[2] Prof. Yee Sin Ang,[1] Prof. Lay Kee Ang,[1] and Prof Liang Jie Wong[2,*]

[1] *Science, Math and Technology, Singapore University of Technology and Design, 8 Somapah Road, Singapore 487372, Singapore*

[2] *School of Electrical and Electronic Engineering, Nanyang Technological University, 50 Nanyang Avenue, Singapore 639798, Singapore*

CONTENTS

S1. Output energy spectrum for different input QEW shapes 2

S2. PINEM quantum interference strength 3
A. Bunching factor and quantum intereference term for a PINEM-modulated QEW 3
B. Bunching factor for a single dominant harmonic 4

S3. Optimal interaction lengths for other dipole moments 6

S4. Approximate expression for the optimal interaction length 7

S5. Derivation of spontaneous emission rate 8
A. Second-quantized operators 8
B. Coupling terms 8
C. Spontaneous emission rate 9
D. Scattering matrix elements 10
E. Spontaneous emission rate from the quantum interference term 12
F. Electron-photon interaction emission rate 13
G. Bound-electron-photon interaction emission rate 13
H. Spontaneous emission rates in the narrow spectral width limit 14
I. Weak coupling regime 14

S6. Neglecting non-energy conserving quantum interference term 15

S7. Quantum interference between distinguishable pathways enabled by shaping goes beyond quantum interference between indistinguishable pathways 16

S8. Conditions for optimizing quantum interference in the free-electron-classical-light field case 17

S9. Experimental Schematic for Free-electron-Bound-electron-Light interaction. 17

* liangjie.wong@ntu.edu.sg

S1. OUTPUT ENERGY SPECTRUM FOR DIFFERENT INPUT QEW SHAPES

In this section, we present supplementary plots for Main Text Figs. 2(g) and 2(h). Specifically, we compare the output quantum electron wavepacket (QEW) energy gain/loss spectrum with and without the effects of quantum interference as a function of coupling strength $\mathcal{G}$ for input QEWs of different shapes (i.e., modulated by different coupling strengths $\mathcal{G}_{\mathrm{mod}}$).

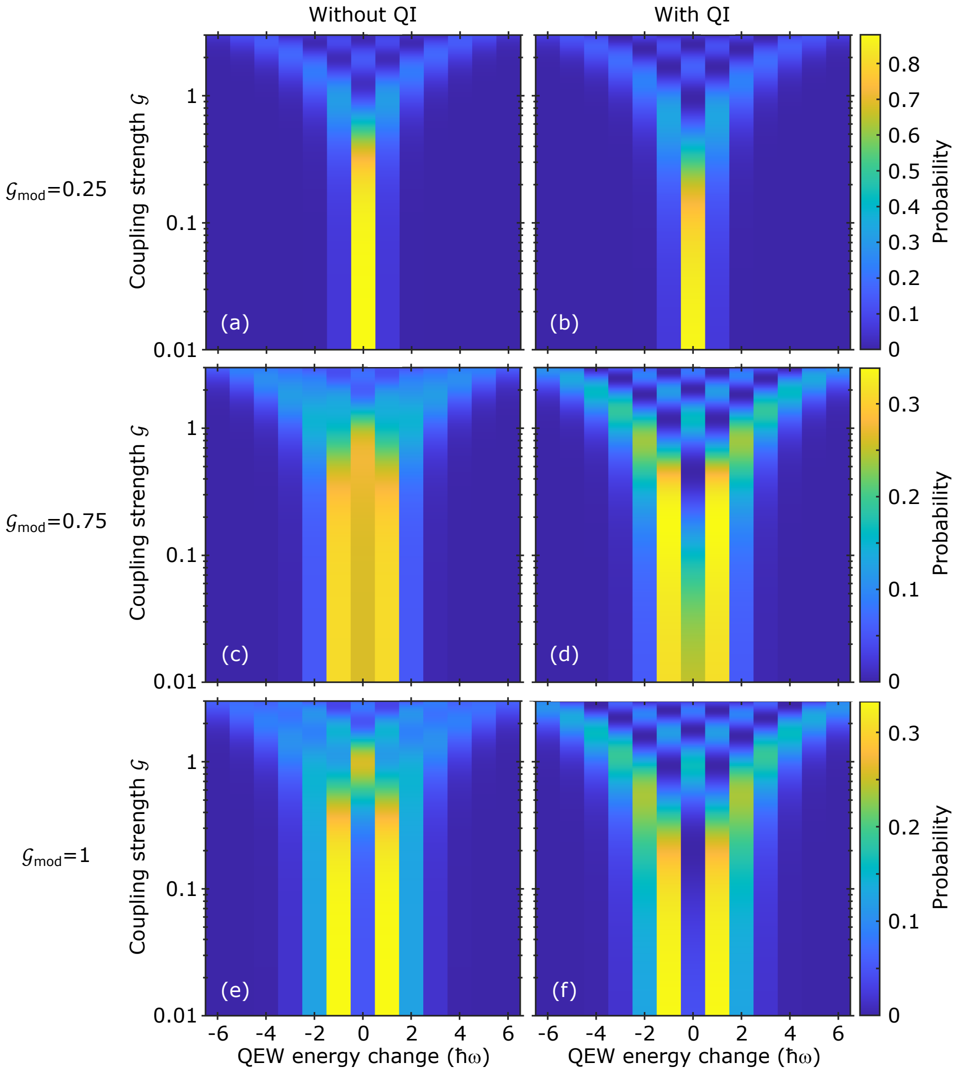

Fig. S1. **Output QEW spectrum as a function of coupling strength $\mathcal{G}$ for different input QEWs shapes.** (a,c,e) show the output spectrum when quantum interference is absent; (b,d,f) show the output spectrum with quantum interference. We consider input QEWs shaped by a coupling strength (a,b) $\mathcal{G}_{\mathrm{mod}} = 0.25$, (c,d) $\mathcal{G}_{\mathrm{mod}} = 0.75$, and $\mathcal{G}_{\mathrm{mod}} = 1$.

We find that for input QEWs that have been weakly modulated ($\mathcal{G}_{\mathrm{mod}} = 0.25$) prior to interaction, as shown in Supplementary Figs. **S1**(a) and (b), the difference between the spectra with quantum interference and without quantum interference is relatively small. We note that the quantum interference contribution still results in a complete suppression of the zero-loss peak at $\mathcal{G} \approx 1$. For input QEWs that have been modulated by stronger coupling strengths of $\mathcal{G}_{\mathrm{mod}} = 0.75$ (Supplementary Figs. **S1**(c) and (d)) and $\mathcal{G}_{\mathrm{mod}} = 1$ (Supplementary Figs. **S1**(e) and (f)), the complete suppression of the zero-loss peak can be achieved with coupling strengths as low as $\mathcal{G} \approx 0.5$ and $\mathcal{G} \approx 0.2$ respectively. As expected, input QEWs that are more strongly modulated prior to interaction result in larger contributions from the quantum interference term.

S2. PINEM QUANTUM INTERFERENCE STRENGTH

Here, we consider spontaneous emission facilitated by a shaped quantum electron wavepacket (QEW). We obtain the shaped QEW by subjecting an unshaped QEW to a photon-induced near-field electron microscopy (PINEM) interaction. In a PINEM interaction, an incoming QEW exchanges an integer number of photons with the the evanescent field of a nanostructure, resulting in the appearance of energy gain/loss peaks separated by the photon energy in the electron spectrum. In Section S2 A, we compute the electron bunching factor for the l-th harmonic of the modulation frequency ω_{mod} for a Gaussian (unmodulated) QEW modulated via the PINEM process. We then derive the corresponding contribution of the quantum interference term to the spontaneous emission rate based on general expressions derived in Section S5. Additionally, we also show in Section S2 B that it suffices to consider only the first harmonic $l = 1$ of the electron bunching factor.

A. Bunching factor and quantum intereference term for a PINEM-modulated QEW

We consider QEWs with wavenumber k_z components tightly-packed about the central longitudinal wavenumber $k_{z,0}$ (i.e., $|k_z - k_{z,0}| \ll k_{z,0}$). We assume that the initial QEW ($z = 0, t = 0$) takes the form of a Gaussian electron wavepacket:

$$\Psi(z=0,t=0) = \frac{1}{\sqrt{2\pi}} \int dk_z \Phi(k_z, t=0), \quad \Phi(k_z, t=0) = \frac{e^{i\phi_0}}{(\pi\sigma_q^2)^{1/4}} e^{-q^2/(2\sigma_q^2)}, \tag{S1}$$

where $q \equiv k_z - k_{z,0}$ is the deviation from the central wavenumber, σ_q is its spread about $k_{z,0}$, and ϕ_0 is the inherently random emission phase. Note that we use wavefunction normalization convention $\int |\Psi(z,t)|^2 dz = \int |\Phi(k_z,t)|^2 dk_z = 1$. During propagation, the wavefunction evolves as

$$\Psi(z,t) = \frac{1}{\sqrt{2\pi}} \int \Phi(k_z,t) e^{ik_z z} dk_z, \tag{S2}$$

where the momentum space wavefunction after propagating for a time t is

$$\Phi(k_z,t) = \frac{e^{i\phi_0}}{(\pi\sigma_q^2)^{1/4}} e^{-q^2/(2\sigma_q^2)} e^{-i\mathcal{E}(q)t/\hbar}. \tag{S3}$$

Consistent with our previous assumption that all k_z are close to the central value, we use the relativistic energy dispersion expanded up to second order in q:

$$\mathcal{E}(q) \approx \gamma_0 m_e c^2 + v_0 \hbar q + \frac{\hbar^2 q^2}{2\gamma_0^3 m_e}. \tag{S4}$$

Assuming the wavepacket drifts a distance L_s between emission and modulation (corresponding to a drift duration $t_s = L_s/v_0$), $\Phi(k_z,t)$ becomes

$$\Phi(k_z,t_s) = \frac{e^{i\phi_0}}{(\pi\sigma_q^2)^{1/4}} e^{-q^2/(2\sigma_q^2)} e^{-i\mathcal{E}(q)L_s/(\hbar v_0)}. \tag{S5}$$

After modulation, and assuming that dispersion is negligible during the modulation, the wavefunction becomes (Ref. [101])

$$\Phi(k_z,t_s) = \frac{e^{i\phi_0}}{(\pi\sigma_q^2)^{1/4}} \sum_{N=-\infty}^{+\infty} e^{iN\phi_{\text{mod}}} J_N(2|g_{\text{mod}}|) \exp\left[-\frac{1}{2\sigma_q^2}\left(q - N\frac{\omega_{\text{mod}}}{v_0}\right)^2 - \frac{iL_s}{\hbar v_0}\mathcal{E}\left(q - N\frac{\omega_{\text{mod}}}{v_0}\right)\right], \tag{S6}$$

where $J_N(\cdot)$ is the Bessel function of the first kind of order N, and $|g_{\text{mod}}|$ is the dimensionless electron-light coupling strength of the modulation stage. The N-th summand describes the momentum deflection as a result of the absorption/emission of N-photons of angular frequency ω_{mod}. Here, ϕ_{mod} is the modulation phase which can be controlled, for instance, using the laser phase. The wavepacket acquires an additional phase when it drifts a distance L_p (corresponding to drift duration $t_p = L_p/v_0$) between modulation and interaction:

$$\Phi(k_z,t_s+t_p) = \frac{e^{i\phi_0}}{(\pi\sigma_q^2)^{1/4}} \exp\left[-\frac{iL_s}{\hbar v_0}\mathcal{E}(q)\right] \sum_{N=-\infty}^{+\infty} e^{iN\phi_{\text{mod}}} J_N(2|g_{\text{mod}}|) \exp\left[-\frac{1}{2\sigma_q^2}\left(q-N\frac{\omega_{\text{mod}}}{v_0}\right)^2 - \frac{iL_s}{\hbar v_0}\mathcal{E}\left(q-N\frac{\omega_{\text{mod}}}{v_0}\right)\right]. \tag{S7}$$

We drop the $t_s + t_p$ dependence in our notation for compactness since we consider the wavefunction only at this time. Additionally, as the momentum space wavefunction only depends on q, we change the dependence: $\Phi(k_z) \to \Phi(q)$.

We now wish to solve for the l-th harmonic of the bunching factor (c.f. Eq. (S56))

$$\langle b\rangle_{l\omega_{\mathrm{mod}}/v_0} = \int \bar{\Phi}(q + l\omega_{\mathrm{mod}}/v_0)\Phi(q)dq, \tag{S8}$$

where we translated the integration variable: $dk_i = dq$. Using Eq. S7, we perform the integration over q, arriving at (Ref. [101])

$$\boxed{\langle b\rangle_{l\omega_{\mathrm{mod}}/v_0} = e^{il[(\pi/2)+(L_p\omega_{\mathrm{mod}}/v_0)-\phi_{\mathrm{mod}}]} e^{-\left(\frac{\sigma_q L_p l\hbar\omega_{\mathrm{mod}}}{2v_0^2\gamma_0^3 m}\right)^2} J_l\left[4|g_{\mathrm{mod}}|\sin\left(L_p\frac{l\hbar\omega_{\mathrm{mod}}^2}{2v_0^3\gamma_0^3 m}\right)\right].} \tag{S9}$$

To find the contribution of the interference term to the spontaneous emission rate, we substitute Eq. (S9) into Eq. (S68) (which we derive in Section S5), yielding

$$\boxed{\begin{aligned}\Gamma^{\mathrm{ap/ep}} =& \frac{\tau}{\hbar}\frac{ev_0\omega_a|\mathbf{d}|}{\epsilon_0 V\omega_{\mathrm{cav}}}|\rho_{eg}^{\mathrm{a}}|\sum_{l\in\mathbb{Z}^+}\left\{e^{-\left(\frac{\sigma_q L_p l\hbar\omega_{\mathrm{mod}}}{2v_0^2\gamma_0^3 m}\right)^2}\cos(\xi_l)J_l\left[4|g_{\mathrm{mod}}|\sin\left(L_p\frac{l\hbar\omega_{\mathrm{mod}}^2}{2v_0^3\gamma_0^3 m}\right)\right]\right.\\ &\left.\times\,\mathrm{sinc}\left[\frac{(\omega_{\mathrm{cav}}-\omega_a)\tau}{2}\right]\mathrm{sinc}\left[\frac{(\beta_0\omega_{\mathrm{cav}}-l\omega_{\mathrm{mod}})\tau}{2}\right]\mathrm{sinc}\left[\frac{(\omega_{\mathrm{cav}}-l\omega_{\mathrm{mod}})\tau}{2}\right]\right\},\end{aligned}} \tag{S10}$$

where the phase of the l-th term is

$$\xi_l = \left(\phi_a - \frac{\omega_{\mathrm{cav}}}{c}z_a - \frac{\pi}{2}\right) + l\left(\frac{\pi}{2} + L_p\frac{\omega_{\mathrm{mod}}}{v_0} - \phi_{\mathrm{mod}}\right). \tag{S11}$$

Here, τ is the interaction time, ϵ_0 is the free space permittivity, V is the mode volume, ω_{cav} is the cavity frequency, and ω_a, $\mathbf{d}$, $\rho_{eg}^{\mathrm{a}} = |\rho_{eg}^{\mathrm{a}}|e^{i\phi_a}$ and z_a are the emission frequency, transition dipole vector, coherence between the excited and ground states and longitudinal position of the two-level atomic (i.e., bound electron) system. Note that to maximize the sum over $l \neq 0$, the phase-matching condition $\xi_l = 0$ should be met, which can be achieved by setting

$$\phi_{\mathrm{mod}} = \frac{\pi}{2} + L_p\frac{\omega_{\mathrm{mod}}}{v_0}, \qquad \frac{\omega_{\mathrm{cav}}}{c}z_a = \phi_a - \frac{\pi}{2}. \tag{S12}$$

Equation (S10) explicitly includes all PINEM parameters that $\langle b\rangle_{l\omega_{\mathrm{mod}}/v_0}$ depend on.

B. Bunching factor for a single dominant harmonic

In this section, we show that the $l = 1$ term in the sum of Eq. (S10) is dominant, allowing us to drop all other terms in deriving an analytical approximation for the quantum interference contribution $\Gamma^{\mathrm{ap/ep}}$. For the specific case of PINEM, which we consider here, it follows that we wish to obtain the value(s) of L_p that maximizes $J_1(\cdot)$. Numerically, we find that $J_1(x)$ reaches the first maxima at $x \approx 1.8411838$, from which we obtain the ideal drift length as

$$L_{p,\mathrm{ideal}} \approx \frac{2v_0^3\gamma^3 m_e}{\hbar\omega_{\mathrm{mod}}^2}\arcsin\Big(1.8411838/4|g_{\mathrm{mod}}|\Big). \tag{S13}$$

Using the expression for $L_{p,\mathrm{ideal}}$, recalling that $\sigma_q \ll \omega_{\mathrm{mod}}/v_0$ must be satisfied for the treatment of QEW momentum states as discrete to be valid, and noting that the arcsin$(\cdot)$ term is of the order of unity, we can make the approximation

$$\exp\left[-\left(\frac{\sigma_q L_{p,\mathrm{ideal}} l\hbar\omega_{\mathrm{mod}}}{2v_0^2\gamma_0^3 m}\right)^2\right] = \exp\left\{-\left[\frac{l\sigma_q}{\omega_{\mathrm{mod}}/v_0}\arcsin\Big(1.8411838/4|g_{\mathrm{mod}}|\Big)\right]^2\right\} \approx 1. \tag{S14}$$

With this approximation, we obtain

$$\begin{aligned}\Gamma^{\mathrm{ap/ep}} \approx& \frac{\tau}{\hbar}\frac{ev_0\omega_a|\mathbf{d}|}{\epsilon_0 V\omega_{\mathrm{cav}}}|\rho_{eg}^{\mathrm{a}}|\cos(\xi_1)J_1\left[4|g_{\mathrm{mod}}|\sin\left(L_p\frac{\hbar\omega_{\mathrm{mod}}^2}{2v_0^3\gamma_0^3 m}\right)\right]\\ &\times\,\mathrm{sinc}\left[\frac{(\omega_{\mathrm{cav}}-\omega_a)\tau}{2}\right]\mathrm{sinc}\left[\frac{(\beta_0\omega_{\mathrm{cav}}-\omega_{\mathrm{mod}})\tau}{2}\right]\mathrm{sinc}\left[\frac{(\omega_{\mathrm{cav}}-\omega_{\mathrm{mod}})\tau}{2}\right].\end{aligned} \tag{S15}$$

Importantly, we find that using the expression for the ideal drift length yields the maximum possible bunching factor magnitude for PINEM:

$$\max\left\{ J_1\left[4|g_{\mathrm{mod}}|\sin\left(L_{p,\mathrm{ideal}}\frac{\hbar\omega_{\mathrm{mod}}^2}{2v_0^3\gamma_0^3 m}\right)\right]\right\} \approx 0.58, \tag{S16}$$

which agrees with the upper limit for bunching factor derived previously (Ref. [15]).

In Fig. **S2**, we compare the total rate $\Gamma = \Gamma^{\mathrm{ap}} + \Gamma^{\mathrm{ep}} + \Gamma^{\mathrm{ap/ep}}$ computed using the approximate expression (Eq. (S15)) for $\Gamma^{\mathrm{ap/ep}}$ with the exact expression Eq. (S10) for various incoming QEW kinetic energies ($\omega_a = \omega_{\mathrm{mod}}$). Here, Γ^{ap} and Γ^{ep} are the bound electron (i.e., atom) and the free electron spontaneous emission rates respectively in the absence of quantum interference; their expressions are also derived in Section S5. We find that the rates computed using the approximate expression (bottom panels) are in excellent agreement with the rate computed with the exact expression (top panels). This indicates that we are justified in considering a single dominant harmonic. We have verified this for other choices of kinetic energies and phases ξ_l. The cyan cross in each panel denotes the value of ω_a and L where the absolute value of the figure of merit, $|\gamma| = |\Gamma^{\mathrm{ap/ep}}/(\Gamma^{\mathrm{ap}} + \Gamma^{\mathrm{ep}})|$, reaches its maximum.

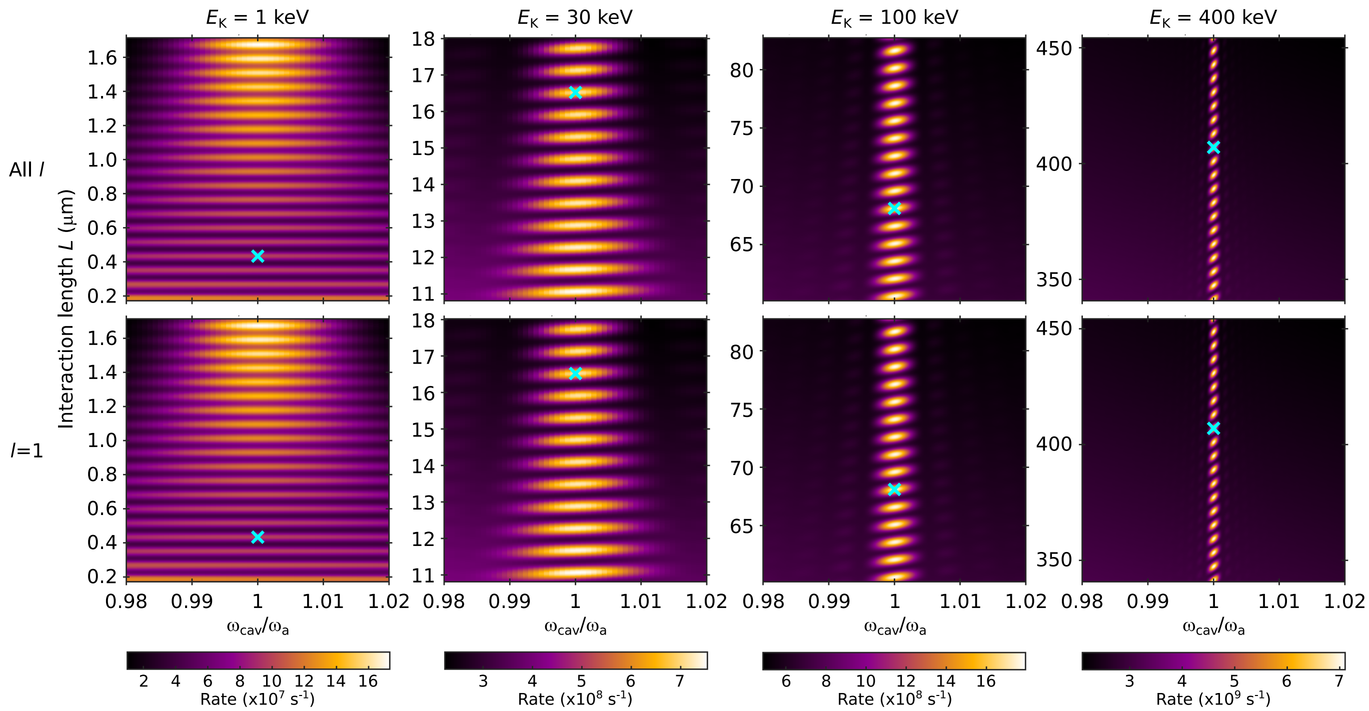

Fig. S2. **Dominance of the first-order Bessel function term in the modulated electron wavefunction for spontaneous emission.** The panels show $\Gamma = \Gamma^{\mathrm{ap}} + \Gamma^{\mathrm{ep}} + \Gamma^{\mathrm{ap/ep}}$, where $\Gamma^{\mathrm{ap/ep}}$ is computed using Eq. (S10) (top panels) and Eq. (S15) (bottom panels), respectively, for various QEW kinetic energies E_{K}. The top panels include all l up to 10: We see excellent agreement between the top panels and the bottom panels, which consider only $l = 1$, indicating the dominance of the $l = 1$ term. In all cases, we consider an incoming QEW modulated with a coupling strength $|g_{\mathrm{mod}}| = 1$ and frequency $\omega_{\mathrm{mod}} = \omega_a$, with the corresponding ideal drift length $L_{p,\mathrm{ideal}}$ given by Eq. (S13). The cyan cross in each panel denotes the ordered pair $(\omega_{\mathrm{cav}}, L)$ corresponding to where the absolute value of the figure of merit, $|\gamma| = |\Gamma^{\mathrm{ap/ep}}/(\Gamma^{\mathrm{ap}} + \Gamma^{\mathrm{ep}})|$, is maximized. The excited and ground states of the bound electron system (Sn-N vacancy) are equally populated initially, although the excellent agreement between Eqs. (S10) and (S15) continues to hold for other initial bound electron states. The phases ξ_l for all cases were chosen according to the optimal condition Eq. (S12), but we have verified that the excellent agreement continues to hold for other choices of ξ_l.

S3. OPTIMAL INTERACTION LENGTHS FOR OTHER DIPOLE MOMENTS

In this section, we plot the optimal interaction length L_{opt} for maximizing the quantum interference contribution as a function of the atomic (i.e., bound electron) emission frequency ω_a and QEW kinetic energy for transition dipole moment magnitudes $|\mathbf{d}| = 10^{-29}$ Cm (Fig. **S3**(a)) and 10^{-30} Cm (Fig. **S3**(b)). We find that dipole moments which are smaller by an order of magnitude require L_{opt} that are larger by about an order of magnitude. However, note that cavity dimensions ranging from $\sim$ 100 nm to $\sim$ 1 mm are still experimentally feasible. In addition, the dependence of the L_{opt} on ω_a and E_{K} remain qualitatively unchanged for all $|\mathbf{d}|$. As $|\mathbf{d}|$ for numerous two-level bound electron systems (e.g., quantum dots, atoms, molecules, ions, superconducting qubits, and crystal defects) are typically of the order of 10^{-29} Cm to 10^{-30} Cm, our results show that significant spontaneous emission modulation rates can be achieved for a wide variety of emitters over practical interaction lengths.

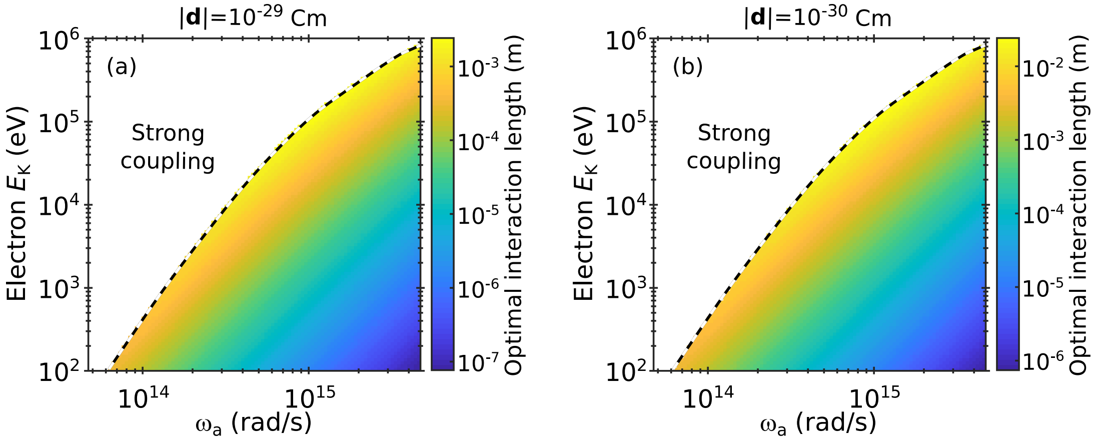

Fig. S3. **Optimal interaction length L_{opt} corresponding to maximum spontaneous emission modulation strength $|\gamma_{\max}|$ as a function of emission angular frequency ω_a and QEW kinetic energy E_{K}.** Here, we plot L_{opt} for transition dipole moments of magnitudes (a) $|\mathbf{d}| = 10^{-29}$ Cm and (b) $|\mathbf{d}| = 10^{-30}$ Cm. Although we find that larger $|\mathbf{d}|$ requires shorter optimal interaction lengths (by about an order of mangitude), cavity lengths ranging from $\sim$ 100 nm to $\sim$ 1 mm are still experimentally feasible. Additionally, the qualitative dependence of L_{opt} on ω_a and E_{K} persists. Since many emitters have dipole moments of the order of 10^{-29} Cm or 10^{-30} Cm, our results show that strong spontaneous emission rate modulation can be achieved in a variety of bound electron systems over practical interaction lengths. We use the same parameters as Main Text Fig. 3(f), where we consider $|\mathbf{d}| = 4.33 \times 10^{-29}$ Cm.

S4. APPROXIMATE EXPRESSION FOR THE OPTIMAL INTERACTION LENGTH

In this section, we derive an approximation for the optimal interaction length $L_{\rm opt}$ where the quantum interference contribution is maximized. Our estimation of $L_{\rm opt}$ involves finding the oscillation envelope of the figure of merit $\gamma = \Gamma^{\rm ap/ep}/(\Gamma^{\rm ap}+\Gamma^{\rm ep})$ as a function of interaction length (or time), and taking its maxima. This yields a ballpark estimate of the exact $L_{\rm opt}$. Using our expressions for $\Gamma^{\rm ap/ep}$, $\Gamma^{\rm ap}$ and $\Gamma^{\rm ep}$ (given by Eq. (S67), which we derive in full in Section S5), we obtain (resonant case)

$$\gamma = 2|\rho^{\rm a}_{eg}||\langle b\rangle|\cos(\xi)\frac{\mathrm{sinc}(\delta_\beta\omega_a\tau/2)}{4\Lambda\frac{1-\mathrm{sinc}(\delta_\beta\omega_a\tau)}{(\delta_\beta\omega_a\tau)^2}+(\rho^{\rm a}_{ee}/\Lambda)}, \tag{S17}$$

where $\Lambda = ev_0/(\omega_a|\mathbf{d}|)$. Noting that the the oscillations of $A\mathrm{sinc}(Ax)$ are always contained within the envelope $\pm 1/x$, we find that the envelope function for Eq. (S17) takes the form (without loss of generality, we consider the positve branch only)

$$\gamma_{\rm env} = \pm\frac{4\Lambda}{\rho^{\rm a}_{ee}}|\rho^{\rm a}_{eg}||\langle b\rangle|\cos(\xi)\frac{l^2}{l^3+\zeta l-\zeta}, \tag{S18}$$

where $\zeta = 4\Lambda^2/\rho^{\rm a}_{ee}$ and $l = \delta_\beta\omega_0\tau$ is the dimensionless interaction length. Taking the first derivative with respect to l, and setting it to zero, we get the depressed cubic equation

$$l^3 - \zeta l + 2\zeta = 0. \tag{S19}$$

Using Cardano's formula, we find that the real root $l_{\rm opt}$ is

$$l_{\rm opt} = C + \frac{\zeta}{3C}, \qquad C = \sqrt[3]{-\zeta+\zeta\sqrt{1-\frac{\zeta}{27}}}. \tag{S20}$$

We note that the interaction length corresponding to $l_{\rm opt}$ lies within one modulation period $\lambda_{\rm SE} = 4\pi v_0/\delta_\beta\omega_a$ of the exact L that maximizes $|\gamma|$. Hence, $l_{\rm opt}$ as an excellent ballpark estimate for the optimal interaction length.

S5. DERIVATION OF SPONTANEOUS EMISSION RATE

In this section, we derive the emission rate for a combined free electron-bound electron-photon system. In Sections S5 A and S5 B, we introduce the second-quantized operators and coupling terms respectively. We then derive the spontaneous emission rate between arbitrary initial and final states in Section S5 C. This is followed by the scattering matrix elements that connect these states up to first-order in perturbation theory in Section S5 D. Having obtained the S-matrix elements, we separately derive the spontaneous emission rates due to the quantum interference term, the free electron-photon interaction term, and the bound electron-photon interaction term (Sections S5 E to S5 G) in the narrow cavity bandwidth limit (Section S5 H). In Sections S5 F and S5 G, we show that our results reduce to known expressions for the spontaneous emission rate from free electron-photon and bound electron-photon interactions respectively. Lastly, we define the limits of the weak coupling regime in Section S5 I.

A. Second-quantized operators

Here, we introduce the notation we adopt for the second quantized operators. For the bound electron state $|s\rangle$ ($s \in \{e, g\}$), the raising and lowering operators, $\hat{\sigma}_+$ and $\hat{\sigma}_-$ respectively, are defined to act in the following ways:

$$\hat{\sigma}_+ |g\rangle = |e\rangle \,, \qquad \hat{\sigma}_- |e\rangle = |g\rangle \,, \tag{S21}$$

and returns zero otherwise. For a free electron, which we model as a quantum electron wavepacket (QEW), in momentum eigenstate $|k\rangle$ (understood to be the longitudinal momentum $\hbar k$ along propagation direction z), $\hat{b}_{\Delta k}$ is the ladder operator that acts as such:

$$\hat{b}_{\Delta k} |k\rangle = |k + \Delta k\rangle \,, \tag{S22}$$

where we define a quantum of momentum deflection Δk such that it can take positive or negative values (i.e., energy gain or loss). For a photon number state $|n\rangle$, we use the usual definitions of the creation and annihilation operators (denoted by $\hat{a}^\dagger$ and $\hat{a}$ respectively):

$$\hat{a} |n\rangle = \sqrt{n} \, |n-1\rangle \,, \qquad \hat{a}^\dagger |n\rangle = \sqrt{n+1} \, |n+1\rangle \,. \tag{S23}$$

B. Coupling terms

Here, we consider the interaction between a QEW, a single longitudinal EM cavity mode of angular frequency ω, and a bound electron modelled as a two-level atom (as described in the main text). The bound electron-photon interaction is described by the Hamiltonian

$$\hat{\mathcal{H}}_{\text{int}}^{\text{ap}} = g_{\text{ap}} \hat{\sigma}_+ \hat{a} + \bar{g}_{\text{ap}} \hat{\sigma}_- \hat{a}^\dagger - \bar{g}_{\text{ap}} \hat{\sigma}_+ \hat{a}^\dagger - g_{\text{ap}} \hat{\sigma}_- \hat{a}, \tag{S24}$$

where g_{ap} is the coupling strength in units of energy. We use an overbar to denote the complex conjugate. The coupling strength takes the following form for a dipole aligned parallel to the field:

$$g_{\text{ap}}(\omega) = i\omega_a |\mathbf{d}| \sqrt{\frac{\hbar}{2\epsilon_0 V \omega}} e^{iqz_a}, \tag{S25}$$

where ω_a is the angular frequency corresponding to the atomic bandgap energy $\mathcal{E}_e - \mathcal{E}_g$, $\hbar$ is the reduced Plank contant, ϵ_0 is the free-space permittivity, V is the mode volume, $q = \omega/c$ is the wavenumber, and z_a is the bound electron system position.

The interaction between the QEW and the photon, which governs effects like PINEM and electron energy-loss spectroscopy, is given by the Hamiltonian (Ref. [27])

$$\hat{\mathcal{H}}_{\text{int}}^{\text{ep}} = \hat{b}_{\Delta k} \Big[g_{\text{ep}}(\Delta k) \hat{a} + \bar{g}_{\text{ep}}(-\Delta k) \hat{a}^\dagger \Big], \tag{S26}$$

where the electron-light coupling strength is

$$g_{\text{ep}}(\Delta k) = -\frac{iev_0}{\omega L} \int_{-L/2}^{L/2} E_{0,z}(\mathbf{r}) e^{-i\Delta k z} dz = ev_0 \sqrt{\frac{\hbar}{2\epsilon_0 V \omega}} \text{sinc}\Big[(q - \Delta k) L/2 \Big]. \tag{S27}$$

Here, e is the elementary charge, L is the interaction length, v_0 is the QEW central velocity, and $E_{0,z}(\mathbf{r}) = i\sqrt{\hbar\omega/(2\epsilon_0 V)}\exp(iqz)$ is the mode profile. We use the convention $\mathrm{sinc}(x) = \sin(x)/x$.

We note that it is possible to rewrite the electron operators in terms of ladder operators. The purpose of this paragraph is to clarify that Eq. S26 is consistent with the displacement operator Hamiltonian used in other works e.g. O. Kfir, *et al.* (Main Text Ref. [27]). Rewriting Eq. S26 explicitly as a sum over all possible momentum change Δk and bringing the operator $\hat{b}_{\Delta k}$ inside:

$$\hat{\mathcal{H}}_{\text{int}}^{\text{ep}} = \sum_{\Delta k} \left[\hat{b}_{\Delta k} g_{\text{ep}}(\Delta k)\hat{a} + \hat{b}_{\Delta k}\bar{g}_{\text{ep}}(-\Delta k)\hat{a}^\dagger\right], \tag{S28}$$

Let us define the ladder operators operating on the free electron:

$$\hat{b}^\dagger |k\rangle \equiv |k + \omega/v_0\rangle = \hat{b}_{\omega/v_0} |k\rangle \tag{S29}$$

$$\hat{b} |k\rangle \equiv |k - \omega/v_0\rangle = \hat{b}_{-\omega/v_0} |k\rangle \tag{S30}$$

Let us restrict the electron to only exchange energy with the photon such that the momentum change satisfies $v_0\Delta k = \omega$. This corresponds to energy conservation when the free electron interacts with a single mode. If we now apply this restriction in the Hamiltonian expression in Eq. S28 and substitute the new operators defined in Eq. S29 and Eq. S30 we obtain:

$$\hat{\mathcal{H}}_{\text{int}}^{\text{ep}} = \hat{b}^\dagger g_{\text{ep}}(\omega/v_0)\hat{a} + \hat{b}\bar{g}_{\text{ep}}(-\omega/v_0)\hat{a}^\dagger \tag{S31}$$

Dropping the ω/v_0 dependence since we've fixed it, we obtain the ladder operator form of the Hamiltonian:

$$\hat{\mathcal{H}}_{\text{int}}^{\text{ep}} = \hat{b}^\dagger g\hat{a} + \hat{b}\bar{g}\hat{a}^\dagger \tag{S32}$$

We also observe that the ladder operators commute as:

$$[\hat{b}^\dagger, \hat{b}] = 0 \tag{S33}$$

Although the interaction Hamiltonian between the QEW and the bound electron – which is the Coulomb interaction – does not contribute to the spontaneous process at the first order in perturbation theory, we state the corresponding Hamiltonian here for completeness (Ref. [101]):

$$\hat{\mathcal{H}}_{\text{int}}^{\text{ea}} = \hat{b}_{\Delta k}\left[g_{eg}^{\text{ea}}(\Delta k)\hat{\sigma}_+ + g_{ge}^{\text{ea}}(\Delta k)\hat{\sigma}_-\right], \tag{S34}$$

where the free electron-bound electron coupling strength is

$$g_{ij}^{\text{ea}}(\Delta k) = \frac{e^2\Delta k}{2\pi\epsilon_0 L} e^{i\Delta k z_a}\Big[-K_1(|\Delta k\Delta r_\perp|)\mathbf{r}_\perp + iK_0(|\Delta k\Delta r_\perp|)\hat{\mathbf{z}}\Big]\cdot \boldsymbol{l}_{ij}. \tag{S35}$$

Here $K_{0,1}(\cdot)$ is the modified Bessel function of the second kind, $\mathbf{r}_\perp = (x, y)$ are the transverse coordinates of the free electron, $\Delta r_\perp$ is the impact parameter that transversally separates the wavepacket from the bound electron, and $\boldsymbol{l}_{ij}$ is the transition dipole length between bound electron states i and j.

The total interaction Hamiltonian is simply the sum of these terms:

$$\hat{\mathcal{H}}_{\text{int}} = \hat{\mathcal{H}}_{\text{int}}^{\text{ap}} + \hat{\mathcal{H}}_{\text{int}}^{\text{ea}} + \hat{\mathcal{H}}_{\text{int}}^{\text{ep}}. \tag{S36}$$

C. Spontaneous emission rate

In this section, we compute the scattering matrix elements given the interaction Hamiltonian Eq. (S36). Consider an initial combined free electron-photon-bound electron state of the form

$$|\text{initial}\rangle = \sum_{k_i,s_i} B_{k_i}^{\text{e}} |k_i\rangle \otimes |0\rangle \otimes B_{s_i}^{\text{a}} |s_i\rangle = \sum_{k_i,s_i} B_{k_i}^{\text{e}} B_{s_i}^{\text{a}} |k_i, 0, s_i\rangle, \tag{S37}$$

where $B_{k_i}^{\text{e}}$ and $B_{s_i}^{\text{a}}$ are the complex amplitudes of finding the initial state containing a free electron at discrete momentum state $|k_i\rangle$ and the bound electron state in $|s_i\rangle$ ($s_i \in \{e, g\}$). As we consider only spontaneous emission,

the initial photon state is the vacuum state $|0\rangle$. As a result of the interaction described by Eq. (S36), $|\text{initial}\rangle$ scatters into a final state $|k_f, n_f, s_f\rangle$ with probability

$$P_{n_f,k_f,s_f} = \left| \sum_{k_i,s_i} B^{\text{e}}_{k_i} B^{\text{a}}_{s_i} C^{n_f,k_f,s_f}_{0,k_i,s_i} \right|^2, \tag{S38}$$

where we have introduced the scattering matrix (S-matrix) element $C^{n_f,k_f,s_f}_{0,k_i,s_i} = \langle n_f, k_f, s_f|\hat{\mathcal{S}}|0, k_i, s_i\rangle$ and the scattering operator

$$\hat{\mathcal{S}} = \hat{\mathcal{T}} \exp\left\{ -\frac{i}{\hbar} \int_{-\infty}^{+\infty} \hat{\mathcal{H}}_{\text{int}}(t)dt \right\}. \tag{S39}$$

Here, $\hat{\mathcal{H}}_{\text{int}}(t) = e^{+i\hat{\mathcal{H}}_0 t/\hbar} \hat{\mathcal{H}}_{\text{int}} e^{-i\hat{\mathcal{H}}_0 t/\hbar}$ is the interaction Hamiltonian in the interaction picture and $\hat{\mathcal{T}}$ denotes time-ordering. We can expand P_{n_f,k_f,s_f} as

$$\begin{aligned} P_{n_f,k_f,s_f} = &\sum_{k_i,s_i} \rho^{\text{a}}_{s_i s_i} \rho^{\text{e}}_{k_i k_i} \left| C^{n_f,k_f,s_f}_{0,k_i,s_i} \right|^2 + \sum_{s_i} \sum_{k_i<K_i} 2\text{Re}\left(\rho^{\text{a}}_{s_i s_i} \rho^{\text{e}}_{k_i K_i} C^{n_f,k_f,s_f}_{0,k_i,s_i} \bar{C}^{n_f,k_f,s_f}_{0,K_i,s_i} \right) + \\ &\sum_{k_i} 2\text{Re}\left(\rho^{\text{a}}_{eg} \rho^{\text{e}}_{k_i k_i} C^{n_f,k_f,s_f}_{0,k_i,e} \bar{C}^{n_f,k_f,s_f}_{0,k_i,g} \right) + \sum_{k_i<K_i} 2\text{Re}\left(\rho^{\text{a}}_{eg} \rho^{\text{e}}_{k_i K_i} C^{n_f,k_f,s_f}_{0,k_i,e} \bar{C}^{n_f,k_f,s_f}_{0,K_i,g} \right), \end{aligned} \tag{S40}$$

where $B^\alpha_m \bar{B}^\alpha_n = \rho_{mn}$ describes the population ($m = n$) and coherence ($m \neq n$) of each system. Here, the superscript refers to the system of interest: $\alpha \in \{\text{e}, \text{a}\}$. Note that in the last 2 terms, we expanded $s_i = e$ and $S_i = g$ for simplicity. The second and third terms can be neglected since none of the interaction matrix elements (given by Eqs. (S47)-(S51)) are zero. Thus, Eq. (S40) reduces to

$$\boxed{P_{n_f,k_f,s_f} = \underbrace{\sum_{k_i,s_i} \rho^{\text{a}}_{s_i s_i} \rho^{\text{e}}_{k_i k_i} \left| C^{n_f,k_f,s_f}_{0,k_i,s_i} \right|^2}_{\text{no QI}} + \underbrace{2\text{Re}\left(\sum_{k_i<K_i} \rho^{\text{a}}_{eg} \rho^{\text{e}}_{k_i K_i} C^{n_f,k_f,s_f}_{0,k_i,e} \bar{C}^{n_f,k_f,s_f}_{0,K_i,g} \right)}_{\text{quantum interference terms}}.} \tag{S41}$$

The first term depends only on the population, while the second describes the contribution arising from quantum interference. We see that non-zero bound electron and QEW coherences, ρ^{a}_{eg} and $\rho^{\text{e}}_{k_i,K_i}$ respectively, are required to observe the effects of quantum interference. Note that this is the same requirement for observing first-order changes in the QEW gain/loss spectra for FEBERI (Ref. [101]). As a result, the incoming QEW needs to contain multiple momentum peaks so that $\rho^{\text{e}}_{k_i,K_i} \neq 0$. Spatio-temporally, this translates to a electron pulse train, whose density varies periodically along the propagation direction in space and time. Therefore, we see that quantum interference arises only when the input QEW and bound electron systems are simultaneously in a superposition of eigenstates. Additionally, note that the eigenstates should be related by a fixed phase relation to ensure nonvanishing contributions upon statistical averaging. We refer to systems that are in a superposition of eigenstates with fixed phase relations as shaped wavefunctions.

D. Scattering matrix elements

In this section, we derive the matrix elements $C^{n_f,k_f,s_f}_{0,k_i,s_i}$. To do this, we consider the weak coupling limit and perturbatively expand the scattering matrix to first order:

$$\hat{\mathcal{S}} \approx \mathbb{I} - \frac{i}{\hbar} \int_{-\infty}^{+\infty} \hat{\mathcal{H}}_{\text{int}}(t)dt = \hat{\mathcal{S}}^{(0)} + \hat{\mathcal{S}}^{(1)}. \tag{S42}$$

We define the j-th-order matrix element as

$$C^{n_f,k_f,s_f(j)}_{0,k_i,s_i} = \langle n_f, k_f, s_f|\hat{\mathcal{S}}^{(j)}|0, k_i, s_i\rangle, \quad j = 0, 1. \tag{S43}$$

Since $n_f \neq 0$ for spontaneous emission, the zeroth order term always vanishes. We then express $C_{0,k_i,s_i}^{n_f,k_f,s_f} \bar{C}_{0,K_i,S_i}^{n_f,k_f,s_f}$ in terms of the interaction matrix elements as

$$\begin{aligned} C_{0,k_i,s_i}^{n_f,k_f,s_f} \bar{C}_{0,K_i,S_i}^{n_f,k_f,s_f} \approx & C_{0,k_i,s_i}^{n_f,k_f,s_f(1)} \bar{C}_{0,K_i,S_i}^{n_f,k_f,s_f(1)} \\ = & \frac{1}{\hbar^2} \langle n_f, k_f, s_f | \hat{\mathcal{H}}_{\text{int}} | 0, k_i, s_i \rangle \langle 0, K_i, S_i | \hat{\mathcal{H}}_{\text{int}}^{\dagger} | n_f, k_f, s_f \rangle \\ & \times \int_{-\infty}^{+\infty} \exp\Big(i \Delta \mathcal{E}_{0,k_i,s_i}^{n_f,k_f,s_f} t/\hbar \Big) dt \int_{-\infty}^{+\infty} \exp\Big(- i \Delta \mathcal{E}_{0,K_i,S_i}^{n_f,k_f,s_f} t/\hbar \Big) dt, \end{aligned} \tag{S44}$$

where we have defined $\Delta \mathcal{E}_{0,k_i,s_i}^{n_f,k_f,s_f} = n_f \hbar\omega + v_0 \hbar (k_f - k_i) \pm \hbar\omega_a$ as the difference between the eigenenergies of the final and initial states. Here, the + (−) branch denotes a bound electron excitation (de-excitation) process. The interaction Hamiltonian matrix element is

$$\begin{aligned} \langle n_f, k_f, s_f | \hat{\mathcal{H}}_{\text{int}} | 0, k_i, s_i \rangle = & g_{eg}^{\text{ea}}(\Delta k) \delta_{n_f,0} \delta_{s_f,s_i+1} \delta_{k_f,k_i+\Delta k} + g_{ge}^{\text{ea}}(\Delta k) \delta_{n_f,0} \delta_{s_f,s_i-1} \delta_{k_f,k_i+\Delta k} + \\ & \bar{g}_{\text{ep}}(-\Delta k) \delta_{n_f,1} \delta_{s_f,s_i} \delta_{k_f,k_i+\Delta k} + \bar{g}_{\text{ap}} \delta_{n_f,1} \delta_{s_f,s_i-1} \delta_{k_f,k_i} - \\ & \bar{g}_{\text{ap}} \delta_{n_f,1} \delta_{s_f,s_i+1} \delta_{k_f,k_i}, \end{aligned} \tag{S45}$$

where $\Delta k = k_f - k_i$, and the Hermitian conjugate is

$$\begin{aligned} \langle 0, K_i, S_i | \hat{\mathcal{H}}_{\text{int}}^{\dagger} | n_f, k_f, s_f \rangle = & \bar{g}_{eg}^{\text{ea}}(\Delta K) \delta_{K_i,k_f-\Delta K} \delta_{n_f,0} \delta_{S_i,s_f-1} + \bar{g}_{ge}^{\text{ea}}(\Delta K) \delta_{K_i,k_f-\Delta K} \delta_{n_f,0} \delta_{S_i,s_f+1} + \\ & g_{\text{ep}}(-\Delta K) \delta_{K_i,k_f-\Delta K} \delta_{n_f,1} \delta_{S_i,s_f} + g_{\text{ap}} \delta_{n_f,1} \delta_{S_i,s_f+1} \delta_{K_i,k_f} - \\ & g_{\text{ap}} \delta_{n_f,1} \delta_{S_i,s_f-1} \delta_{K_i,k_f}, \end{aligned} \tag{S46}$$

where $\Delta K = k_f - K_i$. We multiply each of the 5 terms in Eq. (S45) by Eq. (S46):

$$\begin{aligned} & g_{eg}^{\text{ea}}(\Delta k) \delta_{n_f,0} \delta_{s_f,s_i+1} \delta_{k_f,k_i+\Delta k} \times \langle 0, K_i, S_i | \hat{\mathcal{H}}_{\text{int}}^{\dagger} | n_f, k_f, s_f \rangle \\ = & g_{eg}^{\text{ea}}(\Delta k) \bar{g}_{eg}^{\text{ea}}(\Delta K) \delta_{n_f,0} \delta_{S_i,s_i} \delta_{s_i,g} \delta_{s_f,e} \delta_{K_i,k_f-\Delta K} \delta_{k_f,k_i+\Delta k}, \end{aligned} \tag{S47}$$

$$\begin{aligned} & g_{ge}^{\text{ea}}(\Delta k) \delta_{n_f,0} \delta_{s_f,s_i-1} \delta_{k_f,k_i+\Delta k} \times \langle 0, K_i, S_i | \hat{\mathcal{H}}_{\text{int}}^{\dagger} | n_f, k_f, s_f \rangle \\ = & g_{ge}^{\text{ea}}(\Delta k) \bar{g}_{ge}^{\text{ea}}(\Delta K) \delta_{n_f,0} \delta_{S_i,s_i} \delta_{s_i,e} \delta_{s_f,g} \delta_{k_f,k_i+\Delta k} \delta_{K_i,k_f-\Delta K}, \end{aligned} \tag{S48}$$

$$\begin{aligned} & \bar{g}_{\text{ep}}(-\Delta k) \delta_{n_f,1} \delta_{s_f,s_i} \delta_{k_f,k_i+\Delta k} \times \langle 0, K_i, S_i | \hat{\mathcal{H}}_{\text{int}}^{\dagger} | n_f, k_f, s_f \rangle \\ = & \bar{g}_{\text{ep}}(-\Delta k) g_{\text{ep}}(-\Delta K) \delta_{n_f,1} \delta_{S_i,s_i} \delta_{s_f,s_i} \delta_{k_f,k_i+\Delta k} \delta_{K_i,k_f-\Delta K} + \\ & \bar{g}_{\text{ep}}(-\Delta k) g_{\text{ap}} \delta_{n_f,1} \delta_{S_i,s_i+1} \delta_{s_f,s_i} \delta_{s_i,g} \delta_{k_f,k_i+\Delta k} \delta_{K_i,k_f} - \\ & \bar{g}_{\text{ep}}(-\Delta k) g_{\text{ap}} \delta_{n_f,1} \delta_{S_i,s_i-1} \delta_{s_f,s_i} \delta_{s_i,e} \delta_{k_f,k_i+\Delta k} \delta_{K_i,k_f}, \end{aligned} \tag{S49}$$

$$\begin{aligned} & \bar{g}_{\text{ap}} \delta_{n_f,1} \delta_{s_f,s_i-1} \delta_{k_f,k_i} \times \langle 0, K_i, S_i | \hat{\mathcal{H}}_{\text{int}}^{\dagger} | n_f, k_f, s_f \rangle \\ = & \bar{g}_{\text{ap}} g_{\text{ep}}(-\Delta K) \delta_{n_f,1} \delta_{S_i,s_i-1} \delta_{s_f,g} \delta_{k_f,k_i} \delta_{K_i,k_f-\Delta K} + \\ & \bar{g}_{\text{ap}} g_{\text{ap}} \delta_{n_f,1} \delta_{S_i,s_i} \delta_{s_i,e} \delta_{s_f,g} \delta_{k_f,k_i} \delta_{K_i,k_f}, \end{aligned} \tag{S50}$$

$$\begin{aligned} & - \bar{g}_{\text{ap}} \delta_{n_f,1} \delta_{s_f,s_i+1} \delta_{k_f,k_i} \times \langle 0, K_i, S_i | \hat{\mathcal{H}}_{\text{int}}^{\dagger} | n_f, k_f, s_f \rangle \\ = & - \bar{g}_{\text{ap}} g_{\text{ep}}(-\Delta K) \delta_{n_f,1} \delta_{S_i,s_i+1} \delta_{s_f,e} \delta_{k_f,k_i} \delta_{K_i,k_f-\Delta K} \\ & + \bar{g}_{\text{ap}} g_{\text{ap}} \delta_{n_f,1} \delta_{S_i,s_i} \delta_{s_i,g} \delta_{s_f,e} \delta_{k_f,k_i} \delta_{K_i,k_f}. \end{aligned} \tag{S51}$$

Here, it is understood that $s_i + 1$ refers to excited state "e" if s_i is the ground state "g", and $s_i - 1$ refers to "g" if s_i is "e". Equations (S47) and (S48) do not contribute to the spontaneous emission process as they contain only the FEBERI terms g_{ij}^{ea}. Using these matrix elements, we can compute the transition probabilities to an abitrary final state $|n_f, k_f, s_f\rangle$ through Eq. (S41).

E. Spontaneous emission rate from the quantum interference term

In this section, we derive the spontaneous emission rate arising from the quantum interference term $C^{n_f,k_f,s_f}_{0,k_i,e}\bar{C}^{n_f,k_f,s_f}_{0,K_i,g}$ in Eq. (S41). Because we are interested in a single output photon, we consider only terms proportional to $\delta_{n_f,1}\delta_{S_i,s_i-1}$, where $k_i \neq K_i$, of which there are two:

$$-\bar{g}_{\rm ep}(-\Delta k)g_{\rm ap}\delta_{n_f,1}\delta_{s_f,s_i}\delta_{s_i,e}\delta_{k_f,k_i+\Delta k}\delta_{K_i,k_f}\int_{-\infty}^{+\infty}\exp\Big(i\Delta\mathcal{E}^{n_f,k_f,e}_{0,k_i,e}t/\hbar\Big)dt\int_{-\infty}^{+\infty}\exp\Big(-i\Delta\mathcal{E}^{n_f,k_f,e}_{0,K_i,g}t/\hbar\Big)dt, \quad \text{(S52)}$$

$$\bar{g}_{\rm ap}g_{\rm ep}(-\Delta K)\delta_{n_f,1}\delta_{s_f,g}\delta_{k_f,k_i}\delta_{K_i,k_f-\Delta K}\int_{-\infty}^{+\infty}\exp\Big(i\Delta\mathcal{E}^{n_f,k_f,g}_{0,k_i,e}t/\hbar\Big)dt\int_{-\infty}^{+\infty}\exp\Big(-i\Delta\mathcal{E}^{n_f,k_f,g}_{0,K_i,g}t/\hbar\Big)dt. \quad \text{(S53)}$$

These are lines 3 and 1 of Eqs. (S49) and (S50) respectively. Note that for finite interaction times between $t = -\tau/2$ and $\tau/2$, where $\tau = L/v_0$ is the time-of-flight of the electron over interaction length L, the integral becomes $\int_{-\tau/2}^{+\tau/2} dt\exp(\pm i\Delta\mathcal{E}t/\hbar) = \tau\mathrm{sinc}(\Delta\mathcal{E}\tau/2\hbar)$. Equations (S52) and (S53) then reduce to

$$\begin{aligned}&-\bar{g}_{\rm ep}(-\Delta k)g_{\rm ap}\delta_{s_f,s_i}\delta_{s_i,e}\delta_{k_f,k_i+\Delta k}\delta_{K_i,k_f}\tau^2\mathrm{sinc}\Big[\Delta\mathcal{E}^{1,k_f,e}_{0,k_i,e}\tau/\hbar\Big]\mathrm{sinc}\Big[\Delta\mathcal{E}^{1,k_f,e}_{0,K_i,g}\tau/\hbar\Big]\\ =&-\bar{g}_{\rm ep}(k_i-K_i)g_{\rm ap}\delta_{s_f,s_i}\delta_{s_i,e}\delta_{K_i,k_i+\Delta k}\delta_{K_i,k_f}\tau^2\mathrm{sinc}\Big\{\frac{[\omega-v_0(k_i-K_i)]\tau}{2}\Big\}\mathrm{sinc}\Big[\frac{(\omega+\omega_a)\tau}{2}\Big],\end{aligned} \quad \text{(S54)}$$

and

$$\begin{aligned}&\bar{g}_{\rm ap}g_{\rm ep}(-\Delta K)\delta_{s_f,g}\delta_{k_f,k_i}\delta_{K_i,k_f-\Delta K}\tau^2\mathrm{sinc}\Big[\Delta\mathcal{E}^{1,k_f,g}_{0,k_i,e}\tau/\hbar\Big]\mathrm{sinc}\Big[\Delta\mathcal{E}^{1,k_f,g}_{0,K_i,g}\tau/\hbar\Big]\\ =&\bar{g}_{\rm ap}g_{\rm ep}(K_i-k_i)\delta_{s_f,g}\delta_{k_f,k_i}\delta_{K_i,k_f-\Delta K}\tau^2\mathrm{sinc}\Big[\frac{(\omega-\omega_a)\tau}{2}\Big]\mathrm{sinc}\Big\{\frac{[\omega-v_0(K_i-k_i)]\tau}{2}\Big\},\end{aligned} \quad \text{(S55)}$$

respectively. We neglect Eq. S54, since it is not energy conserving at large L, and is significant only for low-energy QEWs at impractically small L (see SM Section S6). Note that for a QEW modulated at $\omega_{\rm mod}$, we have

$$\sum_{k_i<K_i}\rho^{\rm e}_{k_iK_i} \approx \sum_{l\in\mathbb{Z}^+}\langle b\rangle_{l\omega_{\rm mod}/v_0}, \quad \langle b\rangle_{l\omega_{\rm mod}/v_0} \equiv \int dk_i\Phi(k_i)\bar{\Phi}(k_i+l\omega_{\rm mod}/v_0), \quad \text{(S56)}$$

where we have taken the continuous limit in wavenumber, and defined the bunching factor of the l-th harmonic as $\langle b\rangle_{l\omega_{\rm mod}/v_0}$. Here, $\Phi(k_i)$ is the momentum space wavefunction of the incoming QEW, and the approximation follows from the fact that that the integral is sharply peaked when $l \in \mathbb{Z}$, allowing us to consider only K_i separated from k_i by non-vanishing integer multiples of $\omega_{\rm mod}/v_0$. Equation (S56) tells us that $\rho^{\rm e}_{k_i,K_i}$ is related to the bunching factor $\langle b\rangle$, which in turn describes how the QEW is modulated. For an analytical expression for $\langle b\rangle_{l\omega_{\rm mod}}/v_0$ corresponding to the specific case of an incoming QEW modulated through a photon-induced near-field electron microscopy (PINEM) process, refer to SM Section S2, Eq. (S9). Defining the rate as $\Gamma_{n_f,k_f,s_f} = P_{n_f,k_f,s_f}/\tau$ and multiplying the RHS of Eq. (S55) by the photonic density of states DOS $D_{\rm ph}(\omega)$, we sum over all final bound and free electronic states and integrate over all frequencies to obtain the spontaneous emission rate arising from quantum interference as

$$\boxed{\Gamma^{\rm ap/ep} = \frac{\tau}{\hbar^2}\int 2\mathrm{Re}\Big\{\rho^{\rm a}_{eg}\sum_{l\in\mathbb{Z}^+}\bar{g}_{\rm ap}(\omega)g_{\rm ep}(l\omega_{\rm mod}/v_0)\langle b\rangle_{l\omega_{\rm mod}/v_0}\mathrm{sinc}\Big[\frac{(\omega-l\omega_{\rm mod})\tau}{2}\Big]\mathrm{sinc}\Big[\frac{(\omega-\omega_a)\tau}{2}\Big]\Big\}D_{\rm ph}(\omega)d\omega.} \quad \text{(S57)}$$

Since $\langle b\rangle_{l\omega_{\rm mod}/v_0}$ is a generally complex value, this implies that its phase, which depends on parameters such as drift and dispersion prior to interaction, can be tailored to maximize or minimize quantum interference. Additionally the phase of the bound electron coherence $\rho^{\rm a}_{eg}$ can also result in changes to the interference effect.

F. Electron-photon interaction emission rate

Here, we compute the spontaneous emission rate arising from the photon-free-electron interaction in the absence of the bound electron system. From line 1 of Eq. (S49), we obtain

$$\begin{aligned}
&\frac{1}{\hbar^2}\bar{g}_{\rm ep}(-\Delta k)g_{\rm ep}(-\Delta K)\delta_{S_i,s_i}\delta_{s_f,s_i}\delta_{k_f,k_i+\Delta k}\delta_{K_i,k_f-\Delta K}\frac{1}{\tau}\left[\tau{\rm sinc}(\Delta\mathcal{E}^{1,k_f,s_f}_{0,k_i,s_i}t/\hbar)\right]^2\\
=&\frac{\tau}{\hbar^2}\bar{g}_{\rm ep}(k_i-k_f)g_{\rm ep}(k_i-k_f)\delta_{s_f,s_i}\delta_{k_f,k_i+\Delta k}\delta_{K_i,k_f-\Delta K}{\rm sinc}^2\left\{\frac{[\omega-v_0(k_i-k_f)]\tau}{2}\right\}\\
=&\frac{\tau}{\hbar^2}|g_{\rm ep}(k_i-k_f)|^2\delta_{s_f,s_i}{\rm sinc}^2\left\{\frac{[\omega-v_0(k_i-k_f)]\tau}{2}\right\}.
\end{aligned}\tag{S58}$$

The corresponding emission rate is

$$\Gamma^{el}_{s_f,k_f}=\frac{\tau}{\hbar^2}\sum_{s_i}\rho^{\rm a}_{s_is_i}\int\rho^{\rm e}_{k_i,k_i}|g_{\rm ep}(k_i-k_f)|^2{\rm sinc}^2\left\{\frac{[\omega-v_0(k_i-k_f)]\tau}{2}\right\}dk_i.\tag{S59}$$

Noting that $\sum_{s_i}\rho^{\rm a}_{s_is_i}=1$, multiplying the RHS by the electronic DOS per unit final wavenumber $D_{\rm e}(k_f)=L/2\pi=\tau v_0/2\pi$ and $D_{\rm ph}(\omega)$, and integrating over k_f and ω, we get

$$\Gamma^{\rm ep}=\frac{\tau}{\hbar^2}\int d\omega\int dk_f\int dk_i\rho^{\rm e}_{k_i,k_i}|g_{\rm ep}(k_i-k_f)|^2{\rm sinc}^2\left\{\frac{[\omega-v_0(k_i-k_f)]\tau}{2}\right\}D_{\rm e}(k_f)D_{\rm ph}(\omega).\tag{S60}$$

Since $g_{\rm ep}$ and the sinc terms only depend on $\Delta k=k_f-k_i$, we can change the integral over k_f to an integral over Δk. Noting that $\int dk_i\rho^{\rm e}_{k_i,k_i}=1$, the expression simplifies to

$$\Gamma^{\rm ep}=\frac{\tau}{\hbar^2}\int d\omega\left(\int_{-\infty}^{+\infty}|g_{\rm ep}(-\Delta k)|^2{\rm sinc}^2\left[\frac{(\omega+v_0\Delta k)\tau}{2}\right]d(\Delta k)\right)D_{\rm e}(k_f)D_{\rm ph}(\omega).\tag{S61}$$

Expanding $g_{\rm ep}(\Delta k)$ using Eq. (S27) and solving the integral over Δk using the convolution

$$\int_{-\infty}^{+\infty}{\rm sinc}^2(x){\rm sinc}^2(\xi-x)dx=\left[{\rm sinc}^2*{\rm sinc}^2\right](\xi)=\frac{4\pi[1-{\rm sinc}(\xi)]}{\xi^2},\tag{S62}$$

we find that the free-electron-photon spontaneous emission rate expression simplifies to

$$\boxed{\Gamma^{\rm ep}=\frac{\tau}{\hbar^2}\int\frac{e^2v_0^2\hbar}{2\epsilon_0V\omega}\frac{4[1-{\rm sinc}(\omega\tau\delta_\beta)]}{(\omega\tau\delta_\beta)^2}D_{\rm ph}(\omega)d\omega,}\tag{S63}$$

where $\delta_\beta=|1-\beta_0|$ and $\beta_0=v_0/c$. Equation (S63) reduces to a previously derived result in the limit of long interaction times (Ref. [129]).

G. Bound-electron-photon interaction emission rate

The goal of this section is to show that Eq. (S50) reduces the the standard bound electron (i.e., atomic) spontaneous emission rate in the absence of free electrons. From line 2 of Eq. (S50), we get the bound-electron-light spontaneous emission term:

$$\begin{aligned}
&\frac{1}{\hbar^2}|g_{\rm ap}|^2\delta_{S_i,s_i}\delta_{s_i,e}\delta_{s_f,g}\delta_{k_f,k_i}\delta_{K_i,k_f}\frac{1}{\tau}\left[\tau{\rm sinc}\left(\Delta\mathcal{E}^{1,k_f,s_f}_{0,k_i,s_i}t/\hbar\right)\right]^2\\
=&\frac{\tau}{\hbar^2}|g_{\rm ap}|^2\delta_{s_i,e}\delta_{s_f,g}\delta_{k_f,k_i}\delta_{K_i,k_f}{\rm sinc}^2\left[\frac{(\omega-\omega_a)\tau}{2}\right],
\end{aligned}\tag{S64}$$

and integrating over all final states we find that

$$\boxed{\Gamma^{\rm ap}=\frac{\tau}{\hbar^2}\rho^{\rm a}_{ee}\int|g_{\rm ap}(\omega)|^2{\rm sinc}^2\left[\frac{(\omega-\omega_a)\tau}{2}\right]D_{\rm ph}(\omega)d\omega.}\tag{S65}$$

This expression reduces to the well-known spontaneous emission rate derived from Fermi's Golden rule in the limit of long times.

H. Spontaneous emission rates in the narrow spectral width limit

In this section, we simplify the spontaneous emission rate expressions Eqs. (S57), (S61), and (S65) for the case where the photon density of states $D_{\rm ph}(\omega)$ is described by a Cauchy-Lorentz distribution of spectral width $\Delta\omega_{\rm cav}$:

$$D_{\rm ph}(\omega) = \frac{2}{\pi\Delta\omega_{\rm cav}} \frac{\Delta\omega_{\rm cav}^2}{4(\omega-\omega_{\rm cav})^2 + \Delta\omega_{\rm cav}^2}. \tag{S66}$$

Additionally, we take the narrow cavity bandwidth limit $\Delta\omega_{\rm cav} \to 0$, and the density of states reduces to a delta function: $D_{\rm ph}(\omega) \to \delta(\omega - \omega_{\rm cav})$. Integrating over all frequencies ω, and expanding all coupling terms (given by Eqs. (S25) and (S27)), we obtain the various contributions to the total spontaneous emission rate as

$$\begin{aligned}
\Gamma^{\rm ap} =& \frac{\tau}{\hbar^2} \frac{\hbar\omega_a^2|\mathbf{d}|^2}{2\epsilon_0 V\omega_{\rm cav}} \rho^{\rm a}_{ee} {\rm sinc}^2\left[\frac{(\omega_{\rm cav}-\omega_a)\tau}{2}\right] \\
\Gamma^{\rm ep} =& \frac{\tau}{\hbar^2} \frac{e^2 v_0^2\hbar}{2\epsilon_0 V\omega_{\rm cav}} \frac{4[1-{\rm sinc}(\omega_{\rm cav}\tau\delta_\beta)]}{(\omega_{\rm cav}\tau\delta_\beta)^2} \\
\Gamma^{\rm ap/ep} =& \frac{2\tau}{\hbar^2} \frac{ev_0\hbar\omega_a|\mathbf{d}|}{2\epsilon_0 V\omega_{\rm cav}} {\rm sinc}\left[\frac{(\omega_{\rm cav}-\omega_a)\tau}{2}\right] \times \\
& {\rm Re}\left\{ -i\rho^{\rm a}_{eg} e^{-i\frac{\omega_{\rm cav}}{c}z_a} \sum_{l\in\mathbb{Z}^+} \langle b\rangle_{l\omega_{\rm mod}/v_0} {\rm sinc}\left[\frac{(\beta_0\omega_{\rm cav} - l\omega_{\rm mod})\tau}{2}\right] {\rm sinc}\left[\frac{(\omega_{\rm cav} - l\omega_{\rm mod})\tau}{2}\right]\right\}.
\end{aligned} \tag{S67}$$

Further expressing the l-th harmonic of the bunching factor and bound electron coherence term in their polar forms $\langle b\rangle_{l\omega_{\rm mod}/v_0} = |\langle b\rangle_{l\omega_{\rm mod}/v_0}|e^{i\Psi_b^l}$ and $\rho^{\rm a}_{eg} = |\rho^{\rm a}_{eg}|e^{i\phi_a}$, and collecting all phases of the l-th summand into a single term $\xi_l = \phi_a + \Psi_b^l - \omega_{\rm cav}z_a/c - \pi/2$, we obtain

$$\Gamma^{\rm ap/ep} = \frac{2\tau}{\hbar^2} \frac{ev_0\hbar\omega_a|\mathbf{d}|}{2\epsilon_0 V\omega_{\rm cav}} |\rho^{\rm a}_{eg}| \sum_{l\in\mathbb{Z}^+} |\langle b\rangle_{l\omega_{\rm mod}/v_0}| \cos(\xi_l) {\rm sinc}\left[\frac{(\omega_{\rm cav}-\omega_a)\tau}{2}\right] {\rm sinc}\left[\frac{(\beta_0\omega_{\rm cav} - l\omega_{\rm mod})\tau}{2}\right] {\rm sinc}\left[\frac{(\omega_{\rm cav} - l\omega_{\rm mod})\tau}{2}\right]\Bigg\}. \tag{S68}$$

In the resonant limit $\omega_0 = \omega_a = \omega_{\rm cav} = \omega_{\rm mod}$ – which this study focuses on – the term ${\rm sinc}[(1-l)\omega_0\tau/2]$ in Eq. (S68) is maxmized at $l = 1$. which justifies considering only that term. Furthermore, we can show that the $l = 1$ term dominates even when the resonance condition is not met (see SM Section S2). We thus obtain $\Gamma^{\rm ap/ep}$ as

$$\boxed{\Gamma^{\rm ap/ep} \approx \frac{\tau}{\hbar} \frac{ev_0\omega_a|\mathbf{d}|}{\epsilon_0 V\omega_{\rm cav}} |\rho^{\rm a}_{eg}||\langle b\rangle| \cos(\xi) {\rm sinc}\left[\frac{(\omega_{\rm cav}-\omega_a)\tau}{2}\right] {\rm sinc}\left[\frac{(\beta_0\omega_{\rm cav} - \omega_{\rm mod})\tau}{2}\right] {\rm sinc}\left[\frac{(\omega_{\rm cav} - \omega_{\rm mod})\tau}{2}\right],} \tag{S69}$$

which is Main Text Eq. (3). We have defined $\langle b\rangle_{\omega_{\rm mod}/v_0} = \langle b\rangle$ and $\xi_{l=1} = \xi$ The emission rates from the bound-electron-photon and free-electron-photon interactions are computed using the first two lines of Eq. (S67).

I. Weak coupling regime

In this section, we briefly describe how the maximum interaction times were chosen based on the coupling strengths in the weak coupling limit. For our perturbative expressions to be valid, we impose the following conditions in our study:

$$\frac{\tau}{\hbar}|g_{\rm ap}(\omega_a)| < 0.1, \qquad \frac{\tau}{\hbar}|g_{\rm ep}(\omega_{\rm mod}/v_0)| < 0.1, \tag{S70}$$

where $g_{\rm ap}$ and $g_{\rm ep}$ are given by Eqs. (S25) and (S27) respectively. The maximum interaction duration we consider for the main text is the largest τ that satisfies both conditions.

S6. NEGLECTING NON-ENERGY CONSERVING QUANTUM INTERFERENCE TERM

In this section, we show that the non-energy conserving contribution to the quantum interference term, given by

$$\begin{aligned}\tilde{\Gamma}^{\mathrm{ap/ep}} =& \frac{2\tau}{\hbar^2}\frac{ev_0\hbar\omega_a|\mathbf{d}|}{2\epsilon_0 V\omega_{\mathrm{cav}}}\mathrm{sinc}\left[\frac{(\omega_{\mathrm{cav}}+\omega_a)\tau}{2}\right]\times \\ & \mathrm{Re}\left\{-i\rho^{\mathrm{a}}_{eg}e^{i\frac{\omega_{\mathrm{cav}}}{c}z_a}\sum_{l\in\mathbb{Z}^+}\langle b\rangle_{l\omega_{\mathrm{mod}}/v_0}\mathrm{sinc}\left[\frac{(\beta_0\omega_{\mathrm{cav}}+l\omega_{\mathrm{mod}})\tau}{2}\right]\mathrm{sinc}\left[\frac{(\omega_{\mathrm{cav}}+l\omega_{\mathrm{mod}})\tau}{2}\right]\right\},\end{aligned} \tag{S71}$$

is insignificant for the range of parameters (e.g., QEW kinetic energy, interaction length L) we consider in this work.

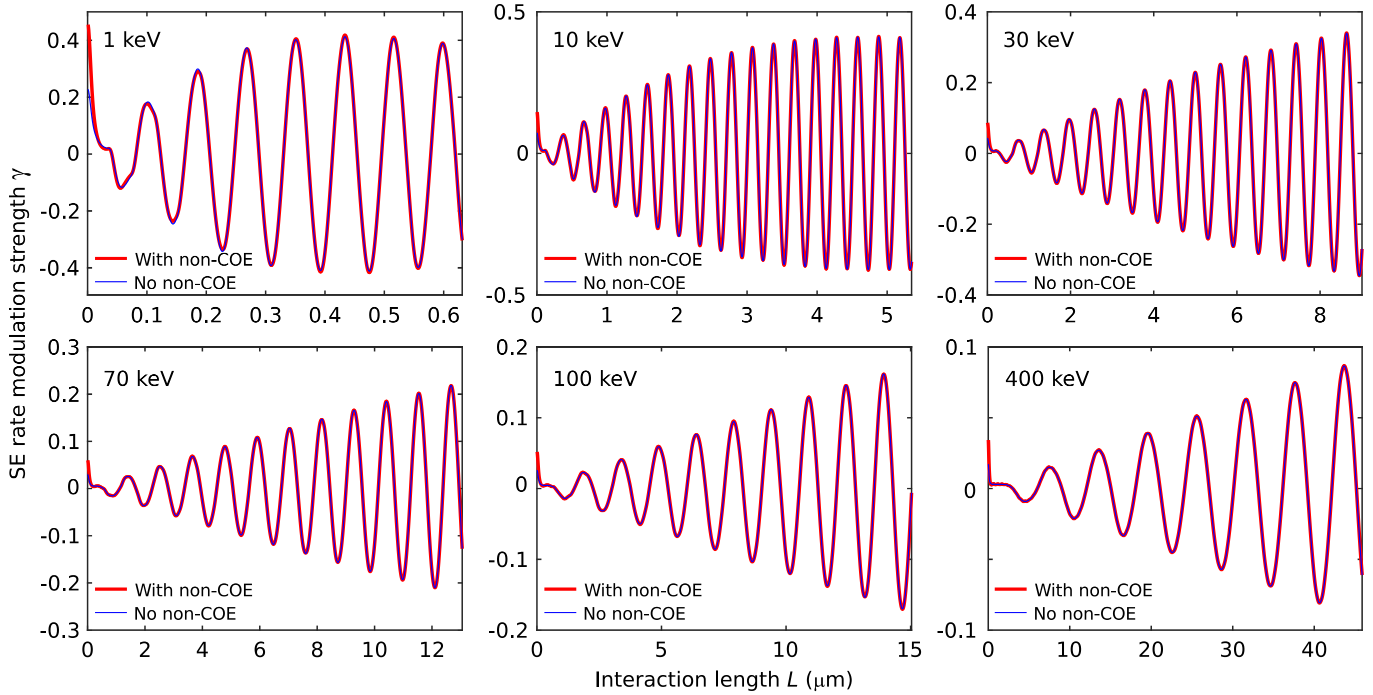

Fig. S4. **Insignificant contribution of the non-energy conservation (non-COE) term $\tilde{\Gamma}^{\mathrm{ap/ep}}$ to the spontaneous emission rate modulation strength γ.** The panels compare $\gamma = (\Gamma^{\mathrm{ap/ep}} + \tilde{\Gamma}^{\mathrm{ap/ep}})/(\Gamma^{\mathrm{ap}} + \Gamma^{\mathrm{ep}})$ (red curves) with $\gamma = \Gamma^{\mathrm{ap/ep}}/(\Gamma^{\mathrm{ap}} + \Gamma^{\mathrm{ep}})$ (blue curves) for various QEW kinetic energies. We see excellent agreement between both curves at longer interaction lengths. The contribution due to $\tilde{\Gamma}^{\mathrm{ap/ep}}$ is only significant for less energetic electrons (≈ 1 keV), and even then, only for extremely short L of the order of ~ 1 nm, which correspond to impractictically small cavity lengths. For higher QEW kinetic energies, the change in γ as a result of $\tilde{\Gamma}^{\mathrm{ap/ep}}$ at small L remains at least an order of magnitude smaller than the peak amplitude of γ (≈ 0.41), which is reached at much larger L. We consider the same QEW parameters as Fig. **S2**, and include all l from -10 to 10.

In Fig. **S4**, we compare $\gamma = (\Gamma^{\mathrm{ap/ep}} + \tilde{\Gamma}^{\mathrm{ap/ep}})/(\Gamma^{\mathrm{ap}} + \Gamma^{\mathrm{ep}})$ (red curves) with $\gamma = \Gamma^{\mathrm{ap/ep}}/(\Gamma^{\mathrm{ap}} + \Gamma^{\mathrm{ep}})$ (blue curves). We compare the figure of merits with and without the non-energy conserving term respectively – as a function of interaction length L for various QEW kinetic energies at $\omega_a = \omega_{\mathrm{cav}} = \omega_{\mathrm{mod}}$. We see that the non-COE (conservation of energy) term only contributes significantly to γ for less energetic electrons (≈ 1 keV to 2 keV) and impractically short cavity lengths (of the order of $L \sim 1$ nm). For more energetic QEWs, the contribution of $\tilde{\Gamma}^{\mathrm{ap/ep}}$ to γ at short times is insignificant (by at least an order of magnitude) as compared to the peak amplitude of γ, which is reached at a large-enough L such that $\tilde{\Gamma}^{\mathrm{ap/ep}} \approx 0$. Hence, we are justified in neglecting the non-COE term in computing the spontaneous emission modulation strengths. We have verified that our conclusions hold for other choices of parameters that fall within the regime of interest of our work.

S7. QUANTUM INTERFERENCE BETWEEN DISTINGUISHABLE PATHWAYS ENABLED BY SHAPING GOES BEYOND QUANTUM INTERFERENCE BETWEEN INDISTINGUISHABLE PATHWAYS

In this section, we emphasize the difference between the quantum interference introduced in this work and previous works on quantum interference between indistinguishable pathways While quantum interference between indistinguishable pathways exists regardless of the shaping (or lack thereof) of the particles involved, the quantum interference discussed in this work requires the initial state of the combined system to be shaped. Specifically it requires that multiple eigenstates that make up the initial state of the combined system transition to the same final state. As an example, let's consider the light-free-electron-bound-electron example we've used in the main text. Since we're concerned purely about spontaneous emission rates, let us fix the initial state of light to be vaccum and the final state of light to correspond to one photon. Consider one such initial state of the combined system such as $|0, k, e\rangle$ corresponding to 0 photons, the free electron with k quanta of energy and e denoting the excited state of the bound electron. One can see that this state can transition to a final state $|1, k, g\rangle$ through many pathways: for example the bound electron could directly exchange energy with the light field (the red path in Fig. **S5** (a)) or have the free electron exchange energy with the light field as well (the blue path in Fig. **S5** (a)). All these pathways interfere and we find quantum path interference to be present in our system. This is interference between indistinguishable yet different pathways. Once we shape our initial interacting systems, we can see that there are multiple eigenstates of the system making up the initial state, that transition to the same final state. For example, the states $|0, k+1, g\rangle$ and $|0, k, e\rangle$ transition to the final state $|1, k, g\rangle$. The interference between the transitions of these different initial eigenstates of the system to the same final state is quantum interference between distinguishable pathways enabled by shaping.

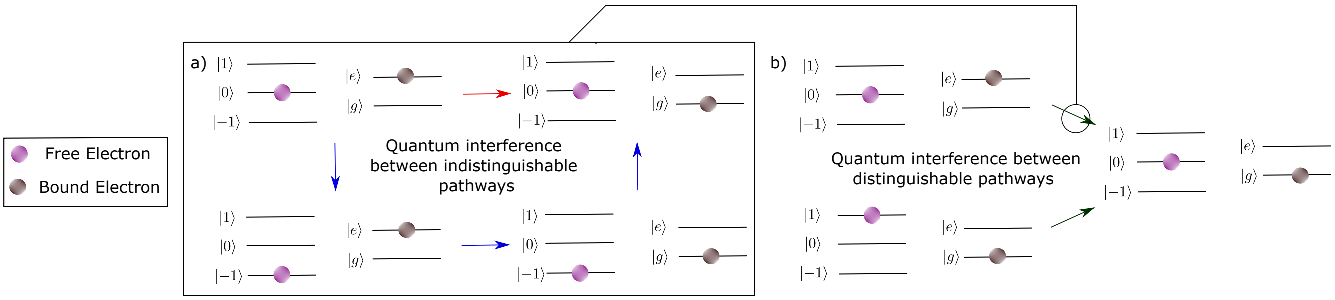

Fig. S5. **Schematic depicting the difference between quantum interference between distinguishable pathways enabled by wave-shaping and quantum interference between indistinguishable pathways** in the context of free-electron-bound-electron emission. Consider the free-electron-bound-electron-light example from the main text. The free electron's possible energy states lie in an energy ladder whereas the bound electron can only take on two states: ground and excited. Here we show the free electron (purple) on the energy ladder with three states labelled ($|-1\rangle$, $|0\rangle$ and $|1\rangle$). The bound electron's (brown) energy spectrum consists of the excited state $|e\rangle$ and the ground state $|g\rangle$. (a) We examine an example of quantum interference between indistinguishable pathways. We start with an excited bound electron and a free electron in the state $|0\rangle$. We can see that for a given final state, where the bound electron transitions to the ground state, there can be many transition pathways that are indistinguishable insofar that their initial states and final states are the same. Shown here are two such pathways: the red path indicates a direct spontaneous emission from the bound electron, the blue path involves emission and absorption from the free electron in the intermediate steps. These pathways can interfere, resulting in quantum interference. This is however distinct from quantum interference between distinguishable pathways enabled by shaping, which occurs due to different input eigenstates transitioning to the same output state. (b) We show an example of quantum interference between distinguishable pathways enabled by shaping. Consider the example where we shape the bound electron to be in a superposition of the ground and excited states. Similarly let us shape the free electron in a superposition of the $|0\rangle$ and $|1\rangle$ states. In this case we see that among the input eigenstates that make up the initial state, we can find eigenstates that transition to the same final state as shown here. These distinguishable transitions from different input eigenstates to the same final state can interfere, giving rise to the quantum interference discussed in this work. Note that both of the pathways discussed in (a) are included in the upper transition pathway in (b).

S8. CONDITIONS FOR OPTIMIZING QUANTUM INTERFERENCE IN THE FREE-ELECTRON-CLASSICAL-LIGHT FIELD CASE

In this section, we define a Figure of Merit to quantify the amount of quantum interference, $\gamma_{\rm QI}$. We then plot the trend of $\gamma_{\rm QI}$ as the shaping parameter $\mathcal{G}_{\rm mod}$ increases, in the free-electron-classical-light field example studied in the main text.

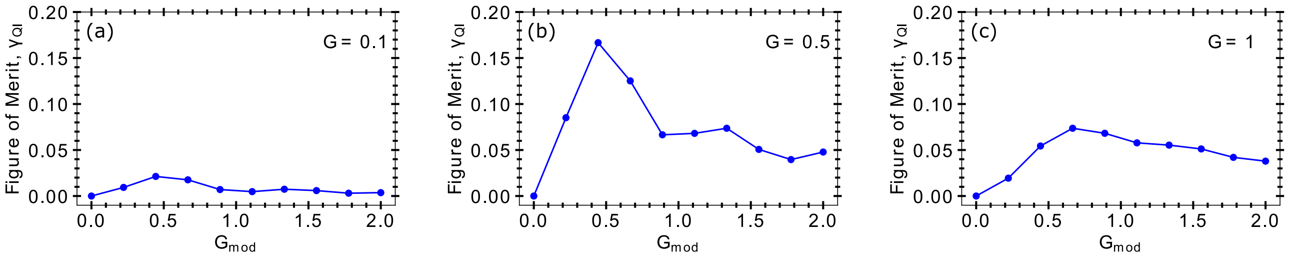

Fig. S6. Plotting figure of merit $\gamma_{\rm QI}$ for quantum interference against increasing shaping parameter $\mathcal{G}_{\rm mod}$ for fixed interaction coupling constant $\mathcal{G}$. We plot the values for $\gamma_{\rm QI}$ for interaction coupling constant values $\mathcal{G} = 0.1, 0.5$ and 1. (a) As expected when $\mathcal{G}$ is very low, we see that $\gamma_{\rm QI}$ is also very low. This is because the electron doesn't exchange energy with the light field very often on account of low coupling strength. (b)(c) We see that at higher values of $\mathcal{G}$, the trend indicates a value of shaping parameter $\mathcal{G}_{\rm mod}$ at which $\gamma_{\rm QI}$ is the highest (for the values of $\mathcal{G}_{\rm mod}$ considered).

We note that here, as in the main text, $\mathcal{G}$ refers to the free-electron coupling constant for the actual interaction (after the shaping). Moreover, $\mathcal{G}_{\rm mod}$ is the modulation coupling constant used to shape the electron in the first place and acts as a shaping parameter. To compute the trend of quantum interference w.r.t. $\mathcal{G}_{\rm mod}$ where we observe high quantum interference, we define a figure of merit, $\gamma_{\rm QI}$. We compute $\gamma_{\rm QI}$ by comparing the statistical distribution of the free electron after the interaction, with and without quantum interference. If the probability of finding the electron in the final state $|K\rangle$ is given by $P(K)_{\rm QI}$ and $P(K)_0$ for the cases with and without quantum interference respectively, we compute $\gamma_{\rm QI}$ as:

$$\gamma_{\rm QI} = \sum_K (P(K)_{\rm QI} - P(K)_0)^2 \tag{S72}$$

This value is computed for a given value of $\mathcal{G}_{\rm mod}$ and coupling constant $\mathcal{G}$. We then vary $\mathcal{G}_{\rm mod}$ while keeping $\mathcal{G}$ fixed and plot the figure of merit $\gamma_{\rm QI}$ as a function of $\mathcal{G}_{\rm mod}$. Shown in Fig. **S6** are the plots for $\mathcal{G} = 0.1$, $\mathcal{G} = 0.5$ and $\mathcal{G} = 1$ for reference.

S9. EXPERIMENTAL SCHEMATIC FOR FREE-ELECTRON-BOUND-ELECTRON-LIGHT INTERACTION.

We consider the setup shown in Fig. **S7**. We choose for our electromagnetic environment, a microcavity with free-electron-light interaction length in the order of 10µm. We then have a two-level system i.e., our bound electron inside the microcavity. Strong bound-electron-light coupling has been shown to occur in semiconductor microcavities [125]. In the schematic we see that the two level system has two energy states that it can access: the ground state denoted by g and the excited state denoted by e. Our two level system has an excitation bandgap frequency of about 10^{15} rad/s and couples to the cavity radiation with coupling strength g_{ap}. Note that this means our assumed interaction length corresponds to the optimal interaction length (from Fig. **S3**) for a Quantum Electron Wavepacket (QEW) of energy in the order of 10 keV. Thus, we fix our QEW energy to be of that order as well. The QEW originates from a nanotip source unmodulated and is subjected to a PINEM modulation to shape it. The PINEM modulation strength can be chosen to tailor the QEW appropriately, here we assume the modulation strength is $G_{mod} = 0.5$. The QEW then travels alongside the microcavity where it exchanges energy with the cavity radiation with coupling strength g_{ep}. We then can measure the QEW spectra through electron spectroscopy measurement. We note that the two level system and QEW do not interact with each other directly. We assume the microcavity to be nearly lossless, with escaping radiation post-interaction being collected by a photodetector for measurement.

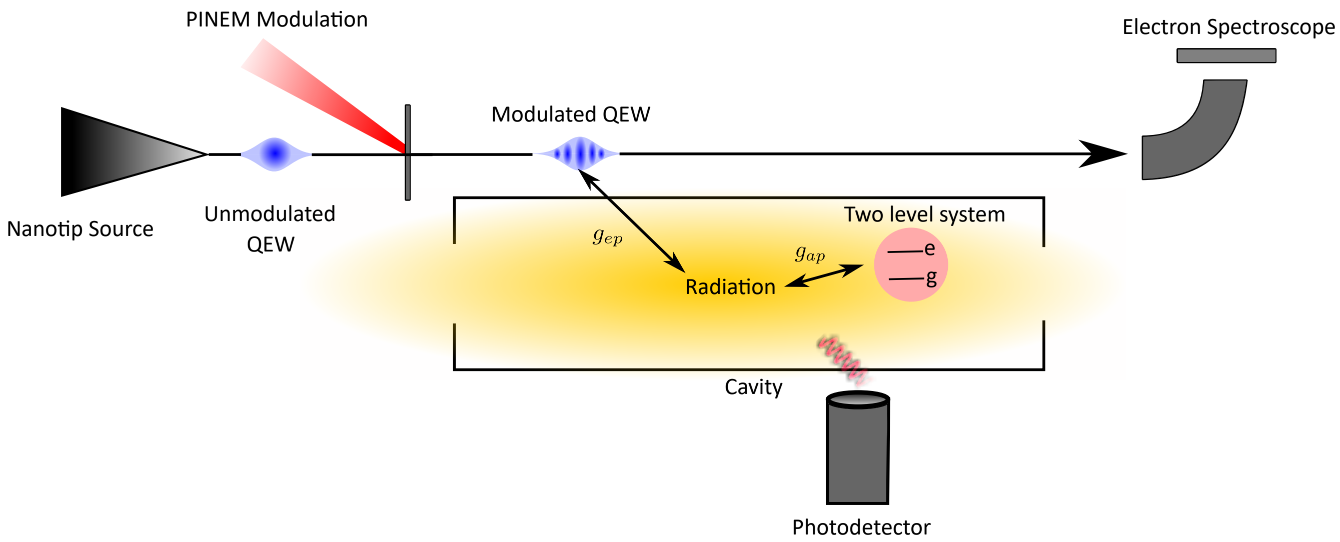

Fig. S7. **Schematic for the free-electron-bound-electron-light interaction.** The free quantum electron wavepacket (QEW) with energy on the order of 100 keV originates from a nanotip source and passes through a PINEM modulation stage. The shaped QEW then passes close to a microcavity with interaction length comparable to 10 µm. The cavity contains a two level system which here is our bound electron system with two possible energy states available to it (ground and excited). The excitation gap frequency of the two level system is on the order of 10^{15} Hz. The two level system-QEW coupling is absent and the cavity is assumed to be nearly lossless. The free electron interacts with a single phase-matched cavity mode as does the two level system. Escaping radiation is captured by a photodetector post interaction. The QEW is measured with the help of a electron detector such as electron energy-loss spectroscopy to examine its spectra.
